# Human Health Effects of Trichloroethylene: Key Findings and Scientific Issues

**DOI:** 10.1289/ehp.1205879

**Published:** 2012-12-18

**Authors:** Weihsueh A. Chiu, Jennifer Jinot, Cheryl Siegel Scott, Susan L. Makris, Glinda S. Cooper, Rebecca C. Dzubow, Ambuja S. Bale, Marina V. Evans, Kathryn Z. Guyton, Nagalakshmi Keshava, John C. Lipscomb, Stanley Barone, John F. Fox, Maureen R. Gwinn, John Schaum, Jane C. Caldwell

**Affiliations:** 1 National Center for Environmental Assessment, and; 2 Office of Children’s Health Protection, U.S. Environmental Protection Agency (EPA), Washington, DC, USA; 3 National Health and Environmental Effects Research Laboratory, U.S. EPA, Research Triangle Park, North Carolina, USA; 4 Office of Pollution Prevention and Toxics, U.S. EPA, Washington, DC, USA; 5 U.S. EPA, Washington, DC, USA (Retired)

**Keywords:** assessment, cancer/tumors, cardiovascular, epidemiology, immunologic response, Integrated Risk Information System (IRIS), meta-analysis, mode of action, physiologically based pharmacokinetic (PBPK) modeling, trichloroethylene

## Abstract

Background: In support of the Integrated Risk Information System (IRIS), the U.S. Environmental Protection Agency (EPA) completed a toxicological review of trichloroethylene (TCE) in September 2011, which was the result of an effort spanning > 20 years.

Objectives: We summarized the key findings and scientific issues regarding the human health effects of TCE in the U.S. EPA’s toxicological review.

Methods: In this assessment we synthesized and characterized thousands of epidemiologic, experimental animal, and mechanistic studies, and addressed several key scientific issues through modeling of TCE toxicokinetics, meta-analyses of epidemiologic studies, and analyses of mechanistic data.

Discussion: Toxicokinetic modeling aided in characterizing the toxicological role of the complex metabolism and multiple metabolites of TCE. Meta-analyses of the epidemiologic data strongly supported the conclusions that TCE causes kidney cancer in humans and that TCE may also cause liver cancer and non-Hodgkin lymphoma. Mechanistic analyses support a key role for mutagenicity in TCE-induced kidney carcinogenicity. Recent evidence from studies in both humans and experimental animals point to the involvement of TCE exposure in autoimmune disease and hypersensitivity. Recent avian and *in vitro* mechanistic studies provided biological plausibility that TCE plays a role in developmental cardiac toxicity, the subject of substantial debate due to mixed results from epidemiologic and rodent studies.

Conclusions: TCE is carcinogenic to humans by all routes of exposure and poses a potential human health hazard for noncancer toxicity to the central nervous system, kidney, liver, immune system, male reproductive system, and the developing embryo/fetus.

Trichloroethylene (TCE) is a chlorinated solvent once widely used as a metal degreaser, chemical intermediate and extractant, and component of some consumer products. Total releases to the environment reported to the U.S. Environmental Protection Agency (EPA) Toxics Release Inventory have declined from > 57 million pounds in 1988 to about 2.4 million pounds in 2010 (U.S. EPA 2012b). Because it has a relatively short half-life, TCE is not commonly detected in biomonitoring surveys, and the percentage of subjects with detectable levels (> 0.1 ng/mL) has declined from about 10% to 1% between samples collected in 1988–1994 and those collected in 2003–2004 (Centers for Disease Control and Prevention 2009; Wu and Schaum 2000]. From a regulatory and environmental-cleanup perspective, TCE has been identified in soil or groundwater at > 700 of approximately 1,300 Superfund hazardous waste sites listed by the U.S. EPA (2011c). Additionally, the U.S. EPA has identified TCE as one of the volatile organic compounds to be regulated as a group in drinking water (U.S. EPA 2010, 2011a) and as one of the priority existing chemicals under review for regulatory action under the Toxic Substances Control Act (U.S. EPA 2012a). Indeed, because of TCE’s continued presence in the environment, most people are likely to have some exposure to the compound through contaminated drinking water, ambient outdoor or indoor air, or, less commonly, contaminated foods.

The U.S. EPA’s Integrated Risk Information System (IRIS) program released an updated human health risk assessment of TCE in September 2011 (U.S. EPA 2011d). This assessment was developed over a period of > 20 years and underwent many stages of both internal and external peer review. Key inputs were recommendations for additional analysis and research from a National Research Council (NRC) panel report reviewing the key scientific issues pertaining to TCE hazard and dose–response assessment (NRC 2006). This report, together with a series of issue papers developed by U.S. EPA scientists (Caldwell and Keshava 2006; Chiu et al. 2006a, 2006b; Keshava and Caldwell 2006; Scott and Chiu 2006), provided the foundation for developing an objective, scientifically rigorous human health risk assessment for TCE. The U.S. EPA’s final assessment also incorporated input from two independent peer reviews by the U.S. EPA’s Science Advisory Board (U.S. EPA SAB 2002, 2011), other federal agencies (U.S. EPA 2009b, 2011b), and the public (U.S. EPA 2009a).

Here we describe key findings and scientific issues addressed in the U.S. EPA’s toxicological review of TCE (U.S. EPA 2011d), covering the following topics: *a*) the role of metabolism in TCE toxicity, which was informed by the development and use of an updated physiologically based pharmacokinetic (PBPK) model; *b*) the carcinogenicity of TCE, including the development of meta-analyses of epidemiologic studies for informing causal inferences, as recommended by the NRC (2006), and analyses of laboratory animal mechanistic and toxicokinetic data contributing to the evaluation of biological plausibility of the epidemiologic data; and *c*) noncancer toxicity related to two end points—immunotoxicity and developmental cardiac toxicity—for which substantial new data have become available. Findings and issues related to other important topics not discussed here (e.g., susceptibility, mixtures/coexposures, and dose–response assessment) have been described previously (e.g., Caldwell JC et al. 2008; NRC 2006; U.S. EPA 2011d).

## Role of Metabolism in TCE Toxicity

**Figure 1:**
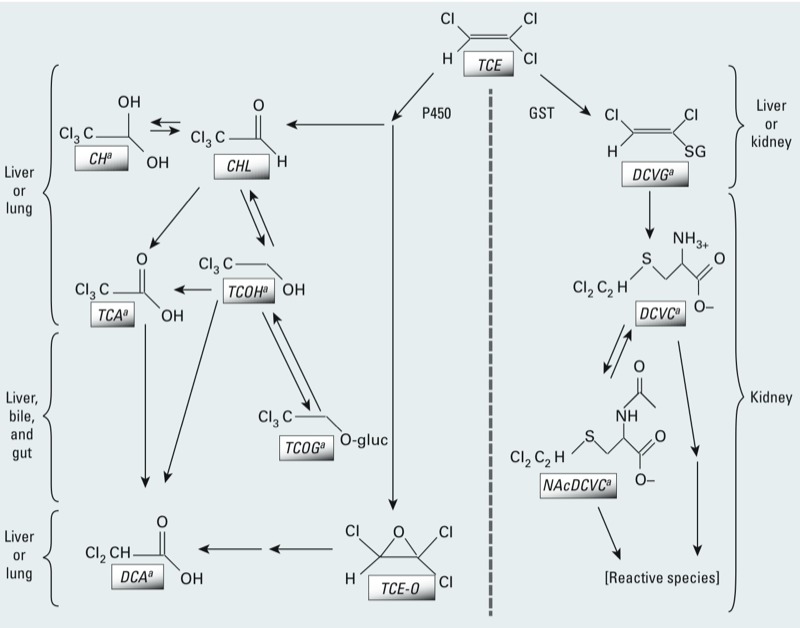
Simplified TCE metabolism scheme. Metabolism of TCE occurs through two main irreversible pathways: oxidation via the microsomal mixed-function oxidase system (i.e., cytochrome P450s; left) and conjugation with GSH by glutathione *S*-transferases (GSTs; right). Oxidation occurs predominantly in the liver, and to a lesser extent in the lung; the first metabolic products are TCE-oxide (TCE‑O), chloral (CHL), and chloral hydrate (CH), with the latter two quickly transformed to trichloroethanol (TCOH; a reversible reaction) and trichloroacetic acid (TCA). TCOH is glucuronidated to form TCOH-glucuronide (TCOG), which undergoes enterohepatic recirculation (excretion in bile with regeneration and reabsorption of TCOH from the gut). TCA and TCOG are excreted in urine. Further metabolism of TCA and TCOH has not been well characterized but may include dichloroacetic acid (DCA) (Lash et al. 2000a). TCE-O may also form DCA, among other species (Cai and Guengerich 1999). TCE conjugation with GSH in the liver or kidney form dichlorovinyl glutathione (DCVG), which is further processed in the kidney, forming the cysteine conjugate *S*-dichlorovinyl-L-cysteine (DCVC). DCVC may be bioactivated by beta-lyase or flavin-containing monooxygenases to reactive species (Anders et al. 1988; Krause et al. 2003; Lash et al. 2003), or (reversibly) undergo *N*-acetylation to the mercapturate *N*-acetyl dichlorovinyl cysteine (NAcDCVC), which is then excreted in urine or sulfoxidated by CYP3A to reactive species (Bernauer et al. 1996; Birner et al. 1993; Werner et al. 1995a, 1995b).

A broad and complex range of relevant information for assessing human health effects of TCE is available. Previous reviews have found TCE to adversely affect the central nervous system (Bale et al. 2011), liver (Bull 2000), kidney (Lash et al. 2000b), immune system (Cooper et al. 2009), and reproductive systems and developing embryo/fetus (NRC 2006). As shown in Figure 1, TCE is metabolized in humans and experimental animal species by both oxidation and glutathione (GSH)-conjugation metabolic pathways, with subsequent production of numerous toxicologically active compounds (Chiu et al. 2006b; Lash et al. 2000a). These include the oxidative metabolites chloral hydrate, trichloroacetic acid (TCA), and dichloroacetic acid, and the GSH conjugation metabolites dichlorovinyl glutathione and dichlorovinyl cysteine. This complex assortment of metabolic compounds is generated from and transported across multiple tissues, making evaluation of mechanistic data especially challenging (Caldwell JC et al. 2008). Liver effects of TCE are thought to result from oxidative metabolites (Buben and O’Flaherty 1985; Bull 2000), whereas effects on kidney are generally associated with metabolites resulting from GSH conjugation (Lash et al. 2000b). The identity of TCE metabolites involved in the induction of other health effects of TCE is less clear, although similarities have been observed between TCE and its oxidative metabolites in the respiratory tract (e.g., Odum et al. 1992) and developmental toxicity (e.g., Johnson et al. 1998a).

Tools such as PBPK models can be very useful for integrating complex toxicokinetic information on absorption, distribution, metabolism, and excretion of TCE and its metabolites. Many PBPK models for TCE have been developed to predict the relationship between external measures of exposure and internal dose measures (Bois 2000a, 2000b; Clewell et al. 2000; Fisher 2000; Hack et al. 2006). Chiu et al. (2009) and Evans et al. (2009) updated and “harmonized” these efforts into a new model for use in the IRIS assessment.

For example, Evans et al. (2009) and Chiu (2011) illustrated the importance of internal dose in investigating mechanisms of TCE toxicity, addressing the key question of whether the TCE metabolite TCA can account for mouse hepatomegaly caused by TCE. They used the TCE PBPK model to compare the hepatomegaly response after TCE administration with the response after direct administration of its metabolite TCA, using the common internal dose measure of TCA liver concentration. If TCA were the only contributor to TCE-induced hepatomegaly, this comparison would show equal changes in liver weight for equal TCA liver concentrations, regardless of whether TCA was the result of TCE metabolism or the result of direct TCA administration. However, as reported by Evans et al. (2009) and Chiu (2011), TCA appears to account for no more than half of the hepatomegaly that resulted from TCE exposure, implying that effects related to TCE exposure beyond those accounted for by TCA are also operative in TCE-induced hepatomegaly.

## Carcinogenicity

*Evaluation of cancer epidemiology for kidney cancer, liver cancer, and non-Hodgkin lymphoma (NHL).* The U.S. EPA conducted a systematic review of 76 human epidemiologic studies on TCE and cancer (Scott and Jinot 2011; U.S. EPA 2011d). Each study was evaluated with respect to explicitly identified characteristics of epidemiologic design and analysis to examine whether chance, bias, or confounding could be alternative explanations for the study’s results. A more in-depth analysis (including meta-analysis) of the epidemiologic studies was conducted for kidney cancer, liver cancer, and NHL. These end points were of *a priori* interest based on the results of a preliminary review of the epidemiologic data and the findings from rodent bioassays of TCE exposure.

**Figure 2:**
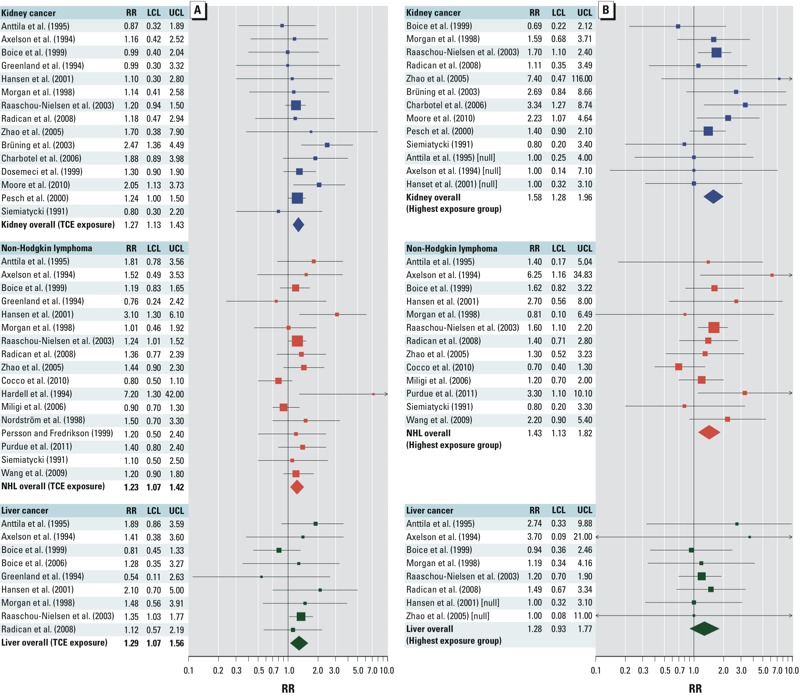
Forest plots from random-effects models of overall (i.e., “ever” or “any”) TCE exposure (*A*) and highest TCE exposure groups (*B*), adapted from Scott and Jinot (2011). Individual study RR (squares) and RRm (diamonds) values are plotted with 95% CIs (LCL, lower confidence limit; UCL, upper confidence limit) for each cancer type. Symbol sizes reflect relative weight of the studies.

*Meta-analysis approach and results.* Meta-analyses can be used to combine underpowered studies, to evaluate effects across the set of studies, and to examine consistency (or heterogeneity) of results. The NRC (2006) identified a number of weaknesses in previous meta-analyses of TCE carcinogenicity, such as subjective assessment of quality and lack of sensitivity analyses. Thus, the U.S. EPA conducted new meta-analyses to support evaluation of the epidemiologic data on TCE (Scott and Jinot 2011; U.S. EPA 2011d). As recommended by the NRC (2006), the U.S. EPA (2011d) *a*) established objective study inclusion criteria; *b*) fit the data to both fixed-effect and random-effects models; *c*) evaluated statistical heterogeneity across the studies; *d*) performed sensitivity analyses examining the influence of individual studies and of different measures of relative risk (RR) from studies presenting alternative estimates (e.g., incidence or mortality); and *e*) conducted tests for potential publication bias (which may occur if positive studies are more likely to be published). Figure 2 presents the meta-analysis summary effect estimates (RRm) from the random-effects models for any TCE exposure (Figure 2A) and for the highest TCE exposure groups (Figure 2B).

*Issues in the interpretation of cancer epidemiologic evidence.* Two additional key issues regarding the U.S. EPA’s interpretation (U.S. EPA 2011d) of the cancer epidemiologic evidence for kidney cancer, NHL, and liver cancer have been raised in peer review and public comments: the modest magnitude of the RRm estimates for the three cancer types, and the role of meta-analysis within a causality determination.

The RRm estimates from the U.S. EPA (2011d) meta-analyses for the three cancer types were modest {e.g., with overall exposure (Figure 2A): 1.27 [95% confidence interval (CI): 1.13, 1.43] for kidney cancer; 1.23 (95% CI: 1.07, 1.42) for NHL, and 1.29 (95% CI: 1.07, 1.56) for liver cancer (Scott and Jinot 2011)}, raising the possibility that the observed associations could be the result of confounding. However, a detailed examination by the U.S. EPA of potential confounding from lifestyle factors or other occupational exposures concluded that confounding was not supported as an alternative explanation for the observed excesses (U.S. EPA 2011d).

For example, although smoking can potentially confound kidney cancer results, several kidney cancer case–control studies included in the meta-analysis (U.S. EPA 2011d) reported associations with TCE exposure even after controlling for smoking in statistical analyses. In addition, if the cohort studies had been confounded by smoking, increased lung cancer risk would be expected. However, increases in lung cancer risk in individual studies were either absent or insufficient to account for the observed excess kidney cancer risk. Overall, after combining studies, RRm estimates for lung cancer were 0.96 (95% CI: 0.76, 1.21) for overall TCE exposure and 0.96 (95% CI: 0.72, 1.27) for the highest exposure groups (Scott and Jinot 2011; U.S. EPA 2011d).

Another key issue is the role of meta-analysis in the overall evaluation of causality. Meta-analysis can provide an objective, quantitative method to increase statistical power and precision because the resultant summary effect estimate is based on multiple studies. Strengths of the meta-analyses (U.S. EPA 2011d) include study identification based on a systematic and transparent review, evaluations of potential publication bias, examinations of the sensitivity of the overall effect to different inputs, and investigations of possible factors responsible for any statistical heterogeneity observed across studies. However, the U.S. EPA’s characterization of the epidemiologic evidence (U.S. EPA 2011d) considered multiple aspects of the data as a whole and did not rely solely on the meta-analysis findings.

*Synthesis of epidemiologic evidence.* Table 1 summarizes the epidemiologic evidence according to the key concepts proposed by Hill (1965). For TCE and kidney cancer, there was convincing evidence of a causal association in humans. Particularly compelling was the consistency of increased RR estimates for kidney cancer across the 15 independent epidemiologic studies of different designs and populations from different countries that met the criteria for inclusion in the meta-analysis (Figure 2). The U.S. EPA (2011d) observed increased RRm estimates for kidney cancer that were robust, not being sensitive to different study or RR inputs. The U.S. EPA (2011d) also found no evidence of heterogeneity among studies or publication bias. The observations of a greater RRm estimate with the highest exposure groups (Figure 2B) and of statistically significant trends between TCE exposure and kidney cancer in two high-quality epidemiologic studies (Charbotel et al. 2006; Moore et al. 2010) support an exposure–response gradient. Finally, potential confounding from smoking or other occupational exposures was unlikely to explain the association of TCE exposure with kidney cancer.

**Table 1:** Primary components for a causality determination based on the epidemiologic database for TCE.

Consideration	Summary of weight of evidence
Consistency of observed association	Strong evidence of consistency for kidney cancer (consistently elevated RRs). Meta‑analysis yielded robust, statistically significant summary RR, with no evidence of heterogeneity or potential publication bias.
	Moderate evidence of consistency for NHL (consistently elevated RRs); RR estimates more variable compared with kidney cancer. Meta-analysis yielded robust, statistically significant summary RR, with some heterogeneity (not statistically significant) and some evidence for potential publication bias.
	Limited evidence of consistency for liver cancer (fewer studies overall, more variable results). Meta-analysis showed no evidence of heterogeneity or potential publication bias, but the statistical significance of the summary estimate depends on the large study by Raaschou-Nielsen et al. (2003).
Strength of observed association	Strength of association is modest. Other known or suspected risk factors (smoking, body mass index, hypertension, or coexposure to other occupational agents such as cutting or petroleum oils) cannot fully explain the observed elevations in kidney cancer RRs. The alternative explanation of smoking was ruled out by the finding of no increased risk of lung cancer. Indirect examination of some specific risk factors for liver cancer or NHL did not suggest confounding as an alternative explanation.
Specificity	Limited evidence suggesting that particular von Hippel-Lindau mutations in kidney tumors may be caused by TCE (Brauch et al. 1999, 2004; Brüning et al. 1997; Nickerson et al. 2008; Schraml et al. 1999); additional research addressing this issue is warranted.
Biological gradient (exposure–response relationship)	Only a few epidemiologic studies examined exposure–response relationships. Studies with well-designed exposure assessments reported a statistically significant trend of increasing risk of kidney cancer (Charbotel et al. 2006; Moore et al. 2010; Zhao et al. 2005) or NHL (Purdue et al. 2011) with increasing TCE exposure. Further support was provided by the meta-analyses; higher summary RR estimates for kidney cancer and NHL were observed for the highest exposure groups than for overall TCE exposure, taking possible reporting bias into account. Liver cancer studies generally had few cases, limiting the ability to assess exposure–response relationships. The meta-analysis for liver cancer did not provide support for a biological gradient (lower summary RR estimate for highest exposure groups than for overall TCE exposure, taking possible reporting bias into account).
Biological plausibility and coherence	TCE metabolism results in reactive, genotoxic, and/or toxicologically active metabolites at target sites in humans and in rodent test species.
	The active GSTT1 enzyme in humans was associated with increased kidney cancer risk, whereas the lack of active enzyme was associated with no increased risk (Moore et al. 2010).
	TCE is carcinogenic in rodents; cancer types with increased incidences include kidney, liver, and lymphohematopoietic cancers.
	A mutagenic mode of action is considered operative for TCE-induced kidney tumors, based on mutagenicity of GSH-conjugation metabolites and the toxicokinetic availability of these metabolites to the target tissue.
	Modes of action are not established for other rodent cancer findings; human relevance is not precluded by any hypothesized modes of action due to inadequate support.
NHL, non-Hodgkin lymphoma. Data from U.S. EPA (2011d).	

The evidence on carcinogenicity from epidemiologic studies of TCE exposure was strong for NHL, although less convincing than for kidney cancer (U.S. EPA 2011d). Of the 17 studies that met the criteria for meta-analysis inclusion, most observed increased RR estimates (Figure 2A). The increased RRm estimate observed in the meta-analysis of NHL and overall TCE exposure was robust because it was not sensitive to different study or RR inputs. However, some heterogeneity among studies was observed, although it was not statistically significant. There was also some evidence of potential publication bias. An exposure–response gradient is supported by observations of a greater RRm estimate with the highest exposure groups (Figure 2B) and of a statistically significant trend between TCE exposure and NHL in a high-quality epidemiologic study (Purdue et al. 2011).

The epidemiologic evidence was more limited for liver cancer, where only cohort studies with small numbers of cases were available (U.S. EPA 2011d). Of the nine studies that met the criteria for meta-analysis inclusion, most reported increased RR estimates (Figure 2A). The U.S. EPA (2011d) observed a statistically significantly increased RRm estimate in their meta-analysis of liver cancer and overall TCE exposure, but the statistical significance depended on the large study by Raaschou-Nielsen et al. (2003). There was no evidence of heterogeneity or publication bias. However, the data available did not support an exposure–response gradient because the RRm estimate for the highest exposure groups was lower than that for overall exposure (Figure 2B) and because none of the available studies reported a statistically significant trend between TCE exposure and liver cancer.

*Experimental animal studies, analysis of mode of action, and toxicokinetic considerations.* There is clear evidence of TCE carcinogenicity in rodents. Particularly notable is the site-concordant finding of TCE-induced kidney tumors in multiple strains and both sexes of rats exposed by inhalation or gavage [Maltoni et al. 1986; National Toxicology Program (NTP) 1988, 1990]. Although the increased incidences were low, they were sometimes statistically significant and were considered biologically significant in light of the very low historical incidences of renal tumors in control rats in various laboratories. There is also site concordance for liver tumors, which were reported in both Swiss and B6C3F_1_ mice (strains with lower and higher background rates of this tumor, respectively), and in both sexes in the latter strain (Maltoni et al. 1986; National Cancer Institute 1976; NTP 1990). The evidence was more limited for TCE-induced lymphohematopoietic cancers in rats and mice (Henschler et al. 1980; Maltoni et al. 1986; NTP 1988, 1990). TCE inhalation bioassays have demonstrated a statistically significant increase in pulmonary tumors in mice (Fukuda et al. 1983; Maltoni et al. 1986) but not other species [i.e., rats and hamsters (Fukuda et al. 1983; Henschler et al. 1980; Maltoni et al. 1986)]. Finally, testicular (interstitial cell and Leydig cell) tumors were significantly increased in Sprague-Dawley rats exposed via inhalation (Maltoni et al. 1986) and Marshall rats exposed via gavage (NTP 1988). In three other tested rat strains, ACI, August, and F344/N, a high (> 75%) control rate of testicular tumors limited the ability to detect a treatment effect, although a positive trend was reported in ACI rats (NTP 1988, 1990). Overall, the rodent cancer data add substantial biological plausibility for TCE carcinogenicity in humans, particularly when combined with the mechanistic data findings.

Table 2 summarizes hypothesized modes of action and mechanistic data informative to the evaluation of TCE’s carcinogenic mode of action for liver, kidney, and other tumors. Mode-of-action analyses can inform judgments regarding the human relevance of animal bioassay results and aid in identifying particularly susceptible populations or life stages (U.S. EPA 2005). For kidney carcinogenicity, the U.S. EPA (2011d) concluded that a mutagenic mode of action is operative for TCE, providing further biological plausibility for the epidemiologic findings of TCE-induced kidney cancer. The identification of the mutagenic metabolites as being derived from the GSH conjugation pathway further suggests increased susceptibility in populations with greater metabolism through this pathway. Consistent with this hypothesis, Moore et al. (2010) found a statistically significant association among TCE-exposed persons with an active GSTT1 (glutathione-*S*-transferase theta-1) enzyme [odds ratio (OR) = 1.88; 95% CI: 1.06, 3.33], but not among those with no GSTT1 activity (OR = 0.93; 95% CI: 0.35, 2.44). Although data are lacking on early-life susceptibility to TCE carcinogenicity, the analysis by Barton et al. (2005) suggested increased susceptibility to cancer from early-life exposures, particularly for chemicals acting through a mutagenic mode of action. For other end points, there are inadequate data to support a particular hypothesized mode of action.

**Table 2:** Selected key mode-of-action hypotheses and support.

End point/hypothesized mode of action	Summary of weight of evidence
Kidney tumors	
Mutagenicity	Data sufficient to conclude a mutagenic mode of action is operative.
GSH conjugation–derived metabolites are produced in the kidney.	Studies demonstrate TCE metabolism via GSH conjugation pathway; availability of metabolites to the kidney in laboratory animals and humans.
Metabolites directly induce mutations in kidney cells, advancing acquisition of critical traits contributing to carcinogenesis.	Predominance of positive genotoxicity data for GSH pathway metabolites in experimental systems.
Cytotoxicity and regenerative proliferation	Data consistent with cytotoxicity contributing to carcinogenesis in rodents, but the evidence is not as strong as that for a mutagenic mode of action.
GSH conjugation–derived metabolites are produced in kidney.	Studies demonstrate TCE metabolism via GSH conjugation pathway; availability of metabolites to the kidney in humans and laboratory animals.
Metabolites directly induce death in kidney cells (cytotoxicity).	Studies demonstrating TCE-induced rare form of nephrotoxicity in laboratory animals; similarity of renal tubular effects induced by TCE and its GSH metabolites. However, cytopathology involves changes in cell and nuclear sizes.
Compensatory cell proliferation occurs to repair damage.	Data linking TCE-induction of proliferation and clonal expansion are lacking.
Clonal expansion of initiated cells occurs, leading to cancer.
Liver tumors	
Mutagenicity	Data are inadequate to support a mutagenic mode of action
Oxidation-pathway–derived metabolites are produced in and/or distributed to the liver.	Studies demonstrate TCE metabolism via oxidative pathway: availability of numerous metabolites to the liver.
Metabolites directly induce mutations in liver, advancing acquisition of critical traits contributing to carcinogenesis.	Strong data for mutagenic potential is CH, but difficult to assess the contributions from CH along with genotoxic and non-genotoxic effects of other oxidative metabolites.
PPARα activation	Data are inadequate to support a PPARα activation mode of action.
Oxidation-pathway–derived PPAR agonist metabolites (TCA and/or DCA) are produced in and/or distributed to the liver.	Studies demonstrate TCE metabolism via oxidative pathway: availability of some metabolites that are PPAR agonists to the liver.
Metabolites activate PPARα in the liver.	Studies demonstrating activation of hepatic PPARα in rodents exposed to TCE and TCA.
Alteration of cell proliferation and apoptosis occurs.	However, inadequate evidence that PPARα is necessary for liver tumors induced by TCE or that hypothesized key events are collectively sufficient for carcinogenesis.
Clonal expansion of initiated cells occurs, leading to cancer.
Other end points and/or modes of action			
Inadequate data to support one or more of the following:		
An identified sequence of key events.		
TCE or metabolites induce key events.		
Key events are individually necessary for inducing the end point.		
Key events are collectively sufficient for inducing the end point.		
Abbreviations: CH, chloral hydrate; DCA, dichloroacetic acid; PPARα, peroxisome proliferator activated receptor α; TCA, trichloroacetic acid. Data from U.S. EPA (2011d).			

The evaluation of TCE carcinogenicity (U.S. EPA 2011d) also considered toxicokinetic data on TCE and metabolites, which are consistent with qualitatively similar absorption, distribution, metabolism, and excretion across species and routes of exposure (Lash et al. 2000a). Mice, rats, and humans all metabolize TCE via the pathways illustrated in Figure 1. Thus, toxicokinetic data support the biological plausibility of TCE carcinogenicity in humans because humans and experimental animals have similar mixtures of TCE and metabolites in target tissues.

Another issue informed by toxicokinetic data is whether TCE carcinogenicity depends on route of exposure, given that the vast majority of the available epidemiologic data are from inhalation exposures to TCE. Because TCE is systemically distributed and undergoes systemic metabolism from all routes of exposure, there is no reason to expect that cancers such as kidney cancer, NHL, or liver cancer, which originate in separate tissues, would be dependent on route of exposure. Also, TCE-induced tumors have been reported in rodents by both the oral and inhalation routes (Maltoni et al. 1986; NTP 1988, 1990). Therefore, conclusions regarding TCE carcinogenicity would apply equally to any exposure route.

*Conclusions as to carcinogenic hazard.* Supported by the analyses described above and following the U.S. EPA’s *Guidelines for Carcinogen Risk Assessment* (U.S. EPA 2005), TCE is characterized as “carcinogenic to humans” by all routes of exposure (U.S. EPA 2011d). This conclusion was based primarily on convincing evidence of a causal association between TCE exposure and kidney cancer in humans. The epidemiologic evidence is strong for NHL, although less convincing than for kidney cancer. Issues increasing the uncertainty in the NHL association include study heterogeneity, potential publication bias, and less evidence for an exposure–response gradient. The epidemiologic evidence was more limited for liver cancer, where only cohort studies with small numbers of cases were available. Finally, animal bioassay, mechanistic, and toxicokinetic data provide further corroboration and biological plausibility to the epidemiologic findings, thus supporting a causal link between TCE exposure and cancer (Table 1).

## Noncancer Toxicity

As part of its evaluation of TCE noncancer toxicity, the U.S. EPA analyzed the available experimental animal, human epidemiologic, and mechanistic studies of TCE. A summary of the relevant studies for each end point is available in Supplemental Material, Table S1 (http://dx.doi.org/10.1289/ehp.1205879). Below we discuss the data pertaining to immunotoxicity and developmental cardiac toxicity, for which there are substantial new experimental and epidemiologic studies (U.S. EPA 2011d), and about which scientific issues have been raised by reviewers or comments. We also provide an overall summary of the hazard conclusions for noncancer toxicity.

*Immunotoxicity.* As recently reviewed by Cooper et al. (2009) and documented in the TCE assessment (U.S. EPA 2011d), the human and laboratory animal studies of TCE and immune-related effects provide strong evidence that TCE exposure increases the risk of autoimmune disease and a specific type of generalized hypersensitivity syndrome. In addition to the epidemiologic studies of specific diseases (e.g., systemic sclerosis), changes in cytokine levels reflecting an inflammatory immune response have been reported in relation to TCE exposure in occupational (Iavicoli et al. 2005) and residential (i.e., infants exposed to TCE in indoor air) (Lehmann et al. 2001, 2002) settings. Also, many case reports have associated a severe hypersensitivity skin disorder, distinct from contact dermatitis and often accompanied by hepatitis, with occupational TCE exposure, with prevalences as high as 13% of workers in the same location (Kamijima et al. 2007, 2008).

Human evidence for autoimmune-related effects is supported by experimental animal studies. Numerous studies have demonstrated TCE-induced progressive, accelerated autoimmune responses in autoimmune-prone mice (reviewed by Cooper et al. 2009). After shorter exposure periods, changes in cytokine levels appear similar to those reported in human studies. Longer exposure periods led to more severe effects, including autoimmune hepatitis, inflammatory skin lesions, and alopecia, that differ from the “normal” expression of autoimmune effects in these mice. TCE-induced autoimmune effects have also been reported in B6C3F_1_ mice, which are not known to have any particular immune-related susceptibility (Gilkeson et al. 2004; Peden-Adams et al. 2006). A treatment-related increase in delayed hypersensitivity response accompanied by hepatic damage has been observed in guinea pigs following intradermal TCE injection (Tang et al. 2002, 2008), and increased hypersensitivity response was reported in mice exposed via drinking water prenatally and postnatally (gestation day 0 through to 8 weeks of age) (Peden-Adams et al. 2006).

There is less evidence regarding a possible role of TCE exposure in immunosuppression. Immunosuppressive effects have been reported in a number of experimental studies in mice and rats [see Supplemental Material, Table S1 (http://dx.doi.org/10.1289/ehp.1205879)]. Reported effects include reduced responses to bacterial challenge in mice (Aranyi et al. 1986; Selgrade and Gilmour 2010) and decreased numbers of antibody-forming cells in rats and developmentally exposed mice (Peden-Adams et al. 2006; Woolhiser et al. 2006).

Overall, the concordance of human and laboratory animal studies and the spectrum of effects (from biomarkers to frank expressions of disease) strongly support the conclusion that TCE causes immunotoxicity, particularly in the form of autoimmune disease and a specific type of severe hypersensitivity skin disorder, with more limited evidence for immunosuppression. Moreover, these findings lend additional biological plausibility to the association between TCE and NHL, as alterations in immune status are associated with increased risk of NHL (Grulich et al. 2007).

*Developmental cardiac toxicity.* The TCE data include a number of epidemiologic and animal toxicity studies that indicate TCE-induced developmental toxicity. Congenital malformations, particularly cardiac defects, have been associated with exposures to TCE and/or its metabolites in both humans and experimental animals [for example studies, see Supplemental Material, Table S1 (http://dx.doi.org/10.1289/ehp.1205879)]. Other TCE-related developmental outcomes observed in both humans and experimental animals include embryonic or fetal mortality, prenatal growth inhibition, and neurological and immunological functional deficits. (see Supplemental Material, Table S1).

As noted by the NRC (2006), the cardiac teratogenicity of TCE has been the focus of considerable study and analysis (Bove et al. 2002; Hardin et al. 2005; Johnson et al. 1998b; Watson et al. 2006). Only geography-based epidemiology studies have evaluated whether there is an association between maternal TCE exposure and cardiac defects in offspring [see Supplemental Material, Table S1 (http://dx.doi.org/10.1289/ehp.1205879)], with some of the studies reporting statistically significant elevations in a variety of cardiac defects [Agency for Toxic Substances and Disease Registry (ATSDR) 2006, 2008; Yauck et al. 2004], and others reporting nonstatistically significant elevations in risk (Bove 1996; Bove et al. 1995; Goldberg et al. 1990). Interpretation of these data has been controversial because many of the studies are limited by small numbers of cases, insufficient exposure characterization, chemical coexposures, and other methodological deficiencies. In addition, these studies aggregate a broad array of TCE-associated cardiac malformations and have inadequate statistical power to identify any particular kind(s) of defect that may be more susceptible to induction by TCE. The NRC (2006) noted that the epidemiologic studies—although limited individually—as a whole showed relatively consistent elevations for cardiac malformations with similar relative effect sizes of 2- to 3-fold, some of which were statistically significant, associated with TCE exposure across multiple studies.

The outcomes of studies in rodents exposed to TCE during gestation show an inconsistent pattern. Some studies identified significant treatment-related increases in the overall incidence of cardiac anomalies at environmentally relevant exposure levels (e.g., Johnson et al. 2003, 2005), whereas others reported no excess cardiac abnormalities at much higher dose levels (e.g., Carney et al. 2006; Fisher et al. 2001). Several methodological factors may contribute to differences across study outcomes, such as the route of administration, test substance purity, test species or strain, timing of dosing or fetal evaluation, procedures used in dissecting and examining fetal hearts, statistical approaches applied to data evaluation, and generally uncharacterized interlaboratory variation.

Other available data providing evidence of TCE cardiac teratogenicity come from avian and *in vitro* mechanistic studies (NRC 2006). For instance, studies in chick embryos reported consistent effects on cardiogenesis (many demonstrating septal and valvular alterations) when TCE was administered during critical stages of heart development (Drake et al. 2006a, 2006b; Loeber et al. 1988; Rufer et al. 2010); these findings are similar to some of the cardiac defects observed in rodent studies following *in utero* TCE exposures (Johnson et al. 2003). The events of cardiac morphogenesis in birds and mammals are similar; both involving mesenchymal cells that form endocardial cushion tissue with subsequent differentiation into septa and valvular structures in the adult heart (NRC 2006). Thus, cultured embryonic chick atrioventricular canal cushion cells have been used to examine chemically induced disruptions in cardiac morphogenesis. In this model, TCE inhibited endothelial separations and mesenchymal cell formation (Boyer et al. 2000; Mishima et al. 2006) or adhesive properties of endocardial cells (Hoffman et al. 2004), either of which could potentially result in septal or valvular malformations. Other TCE-induced effects that may have morphologic consequences in the developing heart include disruption of endothelial oxide synthetase, which has a role in endothelial cell proliferation (Ou et al. 2003), and interference with proteins involved in intercellular Ca^2+^ regulation, which may result in altered blood flow (Caldwell PT et al. 2008, 2010; Collier et al. 2003; Selmin et al. 2008).

Overall, the avian and *in vitro* data substantially increase the biological plausibility for TCE-induced cardiac teratogenesis, and thus strongly support the more limited epidemiologic and *in vivo* rodent data suggesting that TCE induces cardiac teratogenicity. Moreover, mechanistic data support the possibility that multiple modes of action with different targets within the developing heart may be operant in eliciting cardiac malformations, consistent with the reported association between TCE and overall cardiac malformations in the absence of a strong association with any particular type of defect.

*Conclusions as to noncancer hazard.* Table 3 summarizes the evidence for TCE noncancer toxicity across target organs and systems (for additional details, see U.S. EPA 2011d). In addition to the immunotoxicity and developmental cardiac toxicity discussed above, there is strong evidence for TCE-induced neurotoxicity, kidney toxicity, liver toxicity, male reproductive toxicity, and several developmental effects in addition to cardiac toxicity. More limited evidence exists for the toxicity of TCE in the respiratory tract and female reproductive system.

**Table 3:** Key conclusions for TCE noncancer toxicity.

Tissue or organ system	Key conclusions as to human health hazard
Central nervous system	Strong evidence, based on multiple human and experimental animal studies, that TCE causes
	Changes in trigeminal nerve function or morphology
	Impairment of vestibular function.
	Limited evidence, primarily from experimental animal studies, with fewer/more limited human studies, that TCE causes
	Delayed motor function, including during neurodevelopment
	Changes in auditory, visual, and cognitive function or performance.
Kidney	Strong evidence, based on experimental animal studies, a few human studies, and mechanistic studies, that TCE causes nephrotoxicity, particularly in the form of tubular toxicity. Nephrotoxicity is likely mediated primarily through the TCE GSH conjugation metabolite DCVC.
Liver	Limited evidence in humans and strong evidence from experimental animal studies that TCE causes hepatotoxicity but not necrosis. Mice appear to be more sensitive than other experimental species, and hepatotoxicity is likely mediated through oxidative metabolites including, but not exclusively, TCA.
Immune system	Strong evidence, based on multiple human and experimental animal studies, that TCE exposure causes
	Autoimmune disease, including scleroderma
	A specific type of generalized hypersensitivity disorder.
	Limited evidence, primarily from experimental animal studies, with fewer/more limited human studies, that TCE causes immunosuppression.
Respiratory tract	Suggestive evidence, primarily from short-term experimental animal studies, that TCE causes respiratory tract toxicity, primarily in Clara cells.
Reproductive system	Strong evidence, based on multiple human and experimental animal studies, that TCE causes male reproductive toxicity, primarily through effects on the testes, epididymides, sperm, or hormone levels.
	Suggestive evidence, based on few/limited human and experimental animal studies, that TCE causes female reproductive toxicity.
Development	Strong evidence, based on weakly suggestive epidemiologic studies, limited experimental animal studies, and multiple mechanistic studies, that TCE causes fetal cardiac malformations; limited experimental evidence that oxidative metabolites, such as TCA and/or DCA, cause similar effects.
	Limited evidence, primarily from experimental animal studies, with weakly suggestive epidemiologic studies, that TCE causes fetal malformations (in addition to cardiac), prenatal losses, decreased growth or birth weight of offspring, and alterations in immune system function.
Abbreviations: DCVC, S-dichlorovinyl-l-cysteine. Data from U.S. EPA (2011d).	

## Summary

TCE is carcinogenic to humans by all routes of exposure and poses a potential human health hazard for noncancer toxicity to the central nervous system, kidney, liver, immune system, male reproductive system, and the developing embryo/fetus. These conclusions are based on analyses of a broad spectrum of information from thousands of scientific studies and input from numerous scientific reviews. In the last decade, substantial new scientific data on the human health effects of TCE have become available. Moreover, methodologic advancements—such as modeling of TCE toxicokinetics, meta-analyses of epidemiologic studies, and analyses of mechanistic and noncancer hazard information—have improved the scientific rigor and transparency of data interpretation. The approaches and conclusions of the U.S. EPA’s analyses (U.S. EPA 2011d) are consistent with the recommendations of the NRC (2006) and were affirmed by independent peer review through the U.S. EPA’s Science Advisory Board (U.S. EPA SAB 2011). In addition, the International Agency for Research on Cancer (IARC) recently upgraded its carcinogenicity classification of TCE to “carcinogenic to humans” (Guha et al. 2012). Finally, studies on the health effects of TCE continue to report findings similar to those described in the U.S. EPA’s assessment, such as kidney carcinogenicity and toxicity (Karami et al. 2012; Vermeulen et al. 2012), immunotoxicity (Hosgood et al. 2011), and developmental cardiac toxicity (Forand et al. 2012).

## Supplemental Material

(549 KB) PDF
