# Supplementary material for: Human Health Effects of Trichloroethylene: Key Findings and Scientific Issues
Source: Environ Health Perspect. 2012 Dec 18;121(3):303–11. doi: 10.1289/ehp.1205879 (PMC3621199; doi:10.1289/ehp.1205879)
Supplement: (549 KB) PDF [file ehp.1205879.s001.pdf]

Supplemental Material

Human Health Effects of Trichloroethylene: Key Findings and Scientific Issues

Weihsueh A. Chiu[1], Jennifer Jinot[1], Cheryl Siegel Scott[1], Susan L. Makris[1], Glinda S. Cooper[1], Rebecca C. Dzubow[2], Ambuja S. Bale[1], Marina V. Evans[3], Kathryn Z. Guyton[1], Nagalakshmi Keshava[1], John C. Lipscomb[1], Stanley Barone, Jr.[4], John F. Fox[1], Maureen R. Gwinn[1], John Schaum[5], Jane C. Caldwell[1]

[1]National Center for Environmental Assessment, U.S. Environmental Protection Agency (EPA), Washington, D.C., USA

[2]Office of Children's Health Protection, U.S. EPA, Washington, D.C., USA

[3]National Health and Environmental Effects Research Laboratory, U.S. EPA, RTP, NC, USA

[4]Office of Pollution Prevention and Toxics, U.S. EPA, Washington, D.C., USA

[5]Retired

Corresponding Author:

Weihsueh A. Chiu, PhD

National Center for Environmental Assessment – 8623P

U.S. EPA

Two Potomac Yard (North Building)

2733 S. Crystal Drive

Arlington VA 22202

E-mail: chiu.weihsueh@epa.gov

(703) 347-8607 (voice)

(703) 347-8699 (fax)

Supplemental Material, Table S1. Studies relevant to evaluation of TCE non-cancer endpoints.

**Tissue or organ system**	**Endpoint(s)**	**Relevant studies**
Central Nervous System	Changes in trigeminal nerve function or morphology.	Human studies:<br>Barret et al. (1982); Barret et al. (1984); Barret et al. (1987); El Ghawabi et al. (1973); Feldman et al. (1988); Feldman et al. (1992); Kilburn and Warshaw (1993); Kilburn (2002a, b); Mhiri et al. (2004); K Rasmussen et al. (1993a); Ruijten et al. (1991); Triebig et al. (1982); Triebig et al. (1983)<br>Experimental animal studies:<br>Albee et al. (2006); Barret et al. (1991); Barret et al. (1992)
	Impairment of vestibular function.	Human studies:<br>Grandjean et al. (1955); Hirsch et al. (1996); Liu et al. (1988); Rasmussen and Sabroe (1986); Smith (1970); Stewart et al. (1970)<br>Experimental animal studies:<br>Niklasson et al. (1993); Tham et al. (1979); Tham et al. (1984); Umezu et al. (1997)
	Delayed motor function, including during neurodevelopment.	Human studies:<br>Beppu (1968); Gamberale et al. (1976); Gash et al. (2008); Gun et al. (1978); Kilburn and Warshaw (1993); Kilburn and Thornton (1996); Kilburn (2002a, b); K Rasmussen et al. (1993a); Reif et al. (2003); White et al. (1997)<br>Experimental animal studies:<br>Albee et al. (2006); Blossom et al. (2008); Bushnell (1997); Bushnell and Oshiro (2000); Fredriksson et al. (1993); Kishi et al. (1993); Kulig (1987); Moser et al. (1995); Moser et al. (2003); Nunes et al. (2001); Savolainen et al. (1977); Shih et al. (2001); Taylor et al. (1985); Umezu et al. (1997); Waseem et al. (2001); Wolff and Siegmund (1978)
	Changes in auditory, visual, and cognitive function or performance.	Human studies:<br>ATSDR (2002); Burg et al. (1995); Burg and Gist (1999); Chalupa et al. (1960); Gamberale et al. (1976); Kilburn (2002a, b); K Rasmussen et al. (1993c); Reif et al. (2003); Salvini et al. (1971); Stewart et al. (1970); Triebig et al. (1976); Triebig et al. (1977a); Triebig et al. (1977b); Tröster and Ruff (1990); Vernon and Ferguson (1969)<br>Experimental animal studies:<br>Albee et al. (2006); Blain et al. (1994); Boyes et al. (2000); Boyes et al. (2003); Boyes et al. (2005); Crofton et al. (1994); Crofton and Zhao (1997); Fechter et al. (1998); Isaacson and Taylor (1989); Isaacson et al. (1990); Jaspers et al. (1993); Kishi et al. (1993); Kjellstrand et al. (1980); Kulig (1987); Muijser et al. (2000); Ohta et al. (2001); Oshiro et al. (2004); Rebert et al. (1991); Rebert et al. (1993); Rebert et al. (1995); Umezu et al. (1997); Yamamura et al. (1983)

Supplemental Material, Table S1 (continued).

**Tissue or organ system**	**Endpoint(s)**	**Relevant studies**
Kidney	Nephrotoxicity, particularly in the form of tubular toxicity.	Human studies:<br>Bolt et al. (2004); Brüning et al. (1999a); Brüning et al. (1999b); Green et al. (2004); Jacob et al. (2007); Nagaya et al. (1989); Radican et al. (2006); K Rasmussen et al. (1993b); Selden et al. (1993)<br>Experimental animal studies:<br>Chakrabarti and Tuchweber (1988); Dow and Green (2000); Green et al. (1998); Green et al. (2003); Jaffe et al. (1984); NCI (1976); NTP (1988, 1990); Terracini and Parker (1965)<br>Mechanistic studies:<br>Cummings and Lash (2000); Cummings et al. (2000a); Cummings et al. (2000b)
Liver	Hepatotoxicity.	Human studies:<br>Davis et al. (2005); Driscoll et al. (1992); Kamijima et al. (2007); Leigh and Jiang (1993); Nagaya et al. (1993); Neghab et al. (1997); K. Rasmussen et al. (1993); Xu et al. (2009)<br>Experimental animal studies:<br>Bai et al. (1992); Berman et al. (1995); Buben and O'Flaherty (1985); Channel et al. (1998); Dees and Travis (1993); Elcombe et al. (1985); Goel et al. (1992); Goldsworthy and Popp (1987); Kaneko et al. (2000); Kjellstrand et al. (1981); Kjellstrand et al. (1983a); Kjellstrand et al. (1983b); Kumar et al. (2001a); Laughter et al. (2004); Melnick et al. (1987); Merrick et al. (1989); Mirsalis et al. (1989); Nakajima et al. (2000); Neghab et al. (1997); NTP (1990); Nunes et al. (2001); Okino et al. (1991); Ramdhan et al. (2008); Ramdhan et al. (2010); Tao et al. (2000); Tucker et al. (1982); Wang and Stacey (1990); Woolhiser et al. (2006)

Supplemental Material, Table S1 (continued).

Tissue or organ system	Endpoint(s)	Relevant studies
Immune System	Autoimmune disease, including scleroderma.	Human studies:<br>Aryal et al. (2001); Diot et al. (2002); Garabrant et al. (2003); Iavicoli et al. (2005); Lehmann et al. (2001); Lehmann et al. (2002); Maitre et al. (2004); Nietert et al. (1998)<br>Experimental animal studies:<br>Blossom et al. (2004); Blossom and Doss (2007); Blossom et al. (2007); Blossom et al. (2008); Cai et al. (2006); Cai et al. (2008); Gilkeson et al. (2004); Griffin et al. (2000a); Griffin et al. (2000b); Kaneko et al. (2000); Keil et al. (2009); Khan et al. (1995); Peden-Adams et al. (2006); Peden-Adams et al. (2008); White et al. (2000)<br>Mechanistic studies:<br>Wang et al. (2007); Wang et al. (2008)
	A specific type of generalized hypersensitivity disorder.	Human studies:<br>Dai et al. (2004); Huang et al. (2002); Kamijima et al. (2007); Kamijima et al. (2008)<br>Experimental animal studies:<br>Peden-Adams et al. (2006); Tang et al. (2002); Tang et al. (2008)
	Immunosuppression.	Human studies:<br>Lagakos et al. (1986)<br>Experimental animal studies:<br>Aranyi et al. (1986); Blossom and Doss (2007); Blossom et al. (2008); Hobara et al. (1984); Kauffmann et al. (1982); Peden-Adams et al. (2006); Peden-Adams et al. (2008); Sanders et al. (1982); Selgrade and Gilmour (2010); Woolhiser et al. (2006)
Respiratory tract	Respiratory tract toxicity, primarily in Clara cells.	Human studies:<br>Cakmak et al. (2004); Saygun et al. (2007)<br>Experimental animal studies:<br>Green et al. (1997); Kurasawa (1988); Le Mesurier et al. (1980); Lewis et al. (1984); Narotsky et al. (1995); NTP (1990); Odum et al. (1992); Prendergast et al. (1967); Scott et al. (1988); Stewart et al. (1979); Villaschi et al. (1991)

Supplemental Material, Table S1 (continued).

**Tissue or organ system**	**Endpoint(s)**	**Relevant studies**
Reproductive system	Male reproductive toxicity, through effects in the testes, epididymides, on sperm, or on hormone levels.	Human studies:<br>ATSDR (2001); Bardodej and Vyskocil (1956); Chia et al. (1997); Chia et al. (1996); El Ghawabi et al. (1973); Forkert et al. (2003); Goh et al. (1998); Rasmussen et al. (1988); Saihan et al. (1978); Sallmen et al. (1998)<br>Experimental animal studies:<br>Cosby and Dukelow (1992); Forkert et al. (2002); George et al. (1985); George et al. (1986); Kan et al. (2007); Kumar et al. (2000a); Kumar et al. (2000b); Kumar et al. (2001b); Land et al. (1981); Veeramachaneni et al. (2001); Xu et al. (2004); Zenick et al. (1984)<br>Mechanistic studies:<br>DuTeaux et al. (2003); DuTeaux et al. (2004); Xu et al. (2004)
	Female reproductive toxicity.	Human studies:<br>ATSDR (2001); Bardodej and Vyskocil (1956); Sagawa et al. (1973); Sallmén et al. (1995); Zielinski (1973)<br>Experimental animal studies:<br>Berger and Horner (2003); Cosby and Dukelow (1992); George et al. (1985); George et al. (1986); Manson et al. (1984); Wu and Berger (2007)

Supplemental Material, Table S1 (continued).

**Tissue or organ system**	**Endpoint(s)**	**Relevant studies**
Development	Fetal cardiac malformations.	Human studies:<br>ATSDR (2001, 2006a, b, 2008); Bove et al. (1995); Bove (1996); Goldberg et al. (1990); Lagakos et al. (1986); Yauck et al. (2004)<br>Experimental animal studies:<br>Carney et al. (2006); Cosby and Dukelow (1992); Dawson et al. (1990, 1993); Dorfmueller et al. (1979); Epstein et al. (1992); Fisher et al. (2001); Hardin et al. (1981); Healy et al. (1982); PD Johnson et al. (1998); P Johnson et al. (1998); Johnson et al. (2003, 2005); Narotsky and Kavlock (1995); Narotsky et al. (1995); Schwetz et al. (1975); Smith et al. (1989); Smith et al. (1992); Warren et al. (2006)<br>Mechanistic studies:<br>Boyer et al. (2000); Bross et al. (1983); Drake et al. (2006a); Drake et al. (2006b); Loeber et al. (1988); Mishima et al. (2006); Ou et al. (2003); Rufer et al. (2008); Rufer et al. (2010)
	Prenatal losses and decreased growth or birth weight of offspring.	Human studies:<br>ATSDR (1998, 2001, 2006b, 2008); Bove et al. (1995); Bove (1996); Goldberg et al. (1990); Lagakos et al. (1986); Lindbohm et al. (1990); Rodenbeck et al. (2000); Taskinen et al. (1989); Taskinen et al. (1994); Windham et al. (1991)<br>Experimental animal studies:<br>Carney et al. (2006); George et al. (1985); George et al. (1986); Hardin et al. (1981); Healy et al. (1982); Kumar et al. (2000b); Narotsky and Kavlock (1995); Narotsky et al. (1995); Schwetz et al. (1975)

References

Albee R, Spencer P, Johnson K, Bradley G, Marable B, Wilmer J, et al. 2006. Lack of trigeminal nerve toxicity in rats exposed to trichloroethylene vapor for 13 weeks. Int J Toxicol 25:531-540.

Aranyi C, O'Shea W, Graham J, Miller F. 1986. The effects of inhalation of organic chemical air contaminants on murine lung host defenses. Fundam Appl Toxicol 6:713-720.

Aryal B, Khuder S, Schaub E. 2001. Meta-analysis of systemic sclerosis and exposure to solvents. Am J Ind Med 40:271-274.

ATSDR. 1998. Volatile organic compounds in drinking water and adverse pregnancy outcomes: United states marine corps base, camp lejeune, north carolina. Available: http://www.atsdr.cdc.gov/hs/lejeune/.

ATSDR. 2001. Final report: Evaluation of priority health conditions in a community with historical contamination by trichloroethylene. Atlanta, GA:U.S. Department of Health and Human Services.

ATSDR. 2002. Impact of trichloroethylene exposure on oral motor, speech, and hearing in children. Atlanta, GA:U.S. Department of Health and Human Services. Available: http://www.ntis.gov/search/product.aspx?ABBR=PB2004100016.

ATSDR. 2006a. Health consultation: Public health implications of exposures to low-level volatile organic compounds in public drinking water: Endicott area investigation, Broome county, New York. Atlanta, GA:U.S. Department of Health and Human Services. Available: http://www.atsdr.cdc.gov/HAC/pha/EndicottAreaInvestigation113006/EndicottAreaInvestigationHC113006.pdf.

ATSDR. 2006b. Health statistics review: Cancer and birth outcome analysis: Endicott area investigation: Endicott area, town of Union, Broome county, New York. Atlanta, GA:U.S. Department of Health and Humans Services. Available: http://www.atsdr.cdc.gov/HAC/pha/EndicottAreaInvestigation/EndicottHealthStatsReviewHC052606.pdf.

ATSDR. 2008. Health consultation: Health statistics review follow-up: Cancer and birth outcome analysis: Endicott area investigation, Endicott area, town of Union, Broome county, New York. Atlanta, GA:U.S. Department of Health and Human Services. Available: http://www.atsdr.cdc.gov/hac/pha//EndicottAreaInvestigationFollowUp/EndicottAreaHC051508.pdf.

Bai C, Canfield P, Stacey N. 1992. Individual serum bile acids as early indicators of carbon tetrachloride- and chloroform-induced liver injury. Toxicology 75:221-234.

Bardodej Z, Vyskocil J. 1956. The problem of trichloroethylene in occupational medicine: Trichloroethylene metabolism and its effect on the nervous system evaluated as a means of hygienic control. Arch Environ Occup Health 13:581-592.

Barret L, Arsac P, Vincent M, Faure J, Garrel S, Reymond F. 1982. Evoked trigeminal nerve potential in chronic trichloroethylene intoxication. Clin Toxicol 19:419-423.

Barret L, Faure J, Guilland B, Chomat D, Didier B, Debru J. 1984. Trichloroethylene occupational exposure: Elements for better prevention. Int Arch Occup Environ Health 53:283-289.

Barret L, Garrel S, Danel V, Debru J. 1987. Chronic trichloroethylene intoxication: A new approach by trigeminal-evoked potentials? Arch Environ Occup Health 42:297-302.

Barret L, Torch S, Usson Y, Gonthier B, Saxod R. 1991. A morphometric evaluation of the effects of trichloroethylene and dichloroacetylene on the rat mental nerve. Preliminary results. Neurosci Lett 131:141-144.

Barret L, Torch S, Leray C, Sarliève L, Saxod R. 1992. Morphometric and biochemical studies in trigeminal nerve of rat after trichloroethylene or dichloroacetylene oral administration. Neurotoxicology 13:601-614.

Beppu K. 1968. Transmission of the anesthetic agents through the placenta in painless delivery and their effects on newborn infants. Keio J Med 17:81-107.

Berger T, Horner C. 2003. In vivo exposure of female rats to toxicants may affect oocyte quality. Reprod Toxicol 17:273-281.

Berman E, Schlicht M, Moser V, MacPhail R. 1995. A multidisciplinary approach to toxicological screening: I. Systemic toxicity. J Toxicol Environ Health 45:127-143.

Blain L, Lachapelle P, Molotchnikoff S. 1994. Electroretinal responses are modified by chronic exposure to trichloroethylene. Neurotoxicology 15:627-631.

Blossom S, Pumford N, Gilbert K. 2004. Activation and attenuation of apoptosis of cd4+ t cells following in vivo exposure to two common environmental toxicants, trichloroacetaldehyde hydrate and trichloroacetic acid. J Autoimmun 23:211-220.

Blossom S, Doss J. 2007. Trichloroethylene alters central and peripheral immune function in autoimmune-prone mrl(+/+) mice following continuous developmental and early life exposure. J Immunotoxicol 4:129-141.

Blossom S, Doss J, Gilbert K. 2007. Chronic exposure to a trichloroethylene metabolite in autoimmune-prone mrl+/+ mice promotes immune modulation and alopecia. Toxicol Sci 95:401-411.

Blossom S, Doss J, Hennings L, Jernigan S, Melnyk S, James S. 2008. Developmental exposure to trichloroethylene promotes cd4+ t cell differentiation and hyperactivity in association with oxidative stress and neurobehavioral deficits in mrl+/+ mice. Toxicol Appl Pharmacol 231:344-353.

Bolt H, Lammert M, Selinski S, Brüning T. 2004. Urinary alpha1-microglobulin excretion as biomarker of renal toxicity in trichloroethylene-exposed persons. Int Arch Occup Environ Health 77:186-190.

Bove F, Fulcomer M, Klotz J, Esmart J, Dufficy E, Savrin J. 1995. Public drinking water contamination and birth outcomes. Am J Epidemiol 141:850-862.

Bove F. 1996. Public drinking water contamination and birthweight, prematurity, fetal deaths, and birth defects. Toxicol Ind Health 12:255-266.

Boyer A, Finch W, Runyan R. 2000. Trichloroethylene inhibits development of embryonic heart valve precursors in vitro. Toxicol Sci 53:109-117.

Boyes W, Bushnell P, Crofton K, Evans M, Simmons J. 2000. Neurotoxic and pharmacokinetic responses to trichloroethylene as a function of exposure scenario. Environ Health Perspect 108:317-322.

Boyes W, Bercegeay M, Ali J, Krantz T, McGee J, Evans M, et al. 2003. Dose-based duration adjustments for the effects of inhaled trichloroethylene on rat visual function. Toxicol Sci 76:121-130.

Boyes W, Bercegeay M, Krantz T, Evans M, Benignus V, Simmons J. 2005. Momentary brain concentration of trichloroethylene predicts the effects on rat visual function. Toxicol Sci 87:187-196.

Bross G, DiFranceisco D, Desmond M. 1983. The effects of low dosages of trichloroethylene on chick development. Toxicology 28:283-294.

Brüning T, Mann H, Melzer H, Sundberg A, Bolt H. 1999a. Pathological excretion patterns of urinary proteins in renal cell cancer patients exposed to trichloroethylene. Occup Med (Lond) 49:299-305.

Brüning T, Sundberg A, Birner G, Lammert M, Bolt H, Appelkvist E, et al. 1999b. Glutathione transferase alpha as a marker for tubular damage after trichloroethylene exposure. Arch Toxicol 73:246-254.

Buben J, O'Flaherty E. 1985. Delineation of the role of metabolism in the hepatotoxicity of trichloroethylene and perchloroethylene: A dose-effect study. Toxicol Appl Pharmacol 78:105-122.

Burg J, Gist G, Allred S, Rudtke T, Pallos L, Cusack C. 1995. The national exposure registry - morbidity analyses of noncancer outcomes from the trichloroethylene subregistry baseline data. Int J Occup Med Toxicol 4:237 - 257.

Burg J, Gist G. 1999. Health effects of environmental contaminant exposure: An intrafile comparison of the trichloroethylene subregistry. Arch Environ Health 54:231-241.

Bushnell P. 1997. Concentration-time relationships for the effects of inhaled trichloroethylene on signal detection behavior in rats. Fundam Appl Toxicol 36:30-38.

Bushnell P, Oshiro W. 2000. Behavioral components of tolerance to repeated inhalation of trichloroethylene (TCE) in rats. Neurotoxicol Teratol 22:221-229.

Cai P, König R, Khan M, Qiu S, Kaphalia B, Ansari G. 2006. Autoimmune response in mrl+/+ mice following treatment with dichloroacetyl chloride or dichloroacetic anhydride. Toxicol Appl Pharmacol 216:248-255.

Cai P, König R, Boor P, Kondraganti S, Kaphalia B, Khan M, et al. 2008. Chronic exposure to trichloroethene causes early onset of sle-like disease in female mrl +/+ mice. Toxicol Appl Pharmacol 228:68-75.

Cakmak A, Ekici A, Ekici M, Arslan M, Iteginli A, Kurtipek E, et al. 2004. Respiratory findings in gun factory workers exposed to solvents. Respir Med 98:52-56.

Carney E, Thorsrud B, Dugard P, Zablotny C. 2006. Developmental toxicity studies in crl:Cd (sd) rats following inhalation exposure to trichloroethylene and perchloroethylene. Birth Defects Res B Dev Reprod Toxicol 77:405-412.

Chakrabarti S, Tuchweber B. 1988. Studies of acute nephrotoxic potential of trichloroethylene in fischer 344 rats. J Toxicol Environ Health 23:147-158.

Chalupa B, Synková J, Ševčík M. 1960. The assessment of electroencephalographic changes and memory disturbances in acute intoxications with industrial poisons. Br J Ind Med 17:238-241.

Channel S, Latendresse J, Kidney J, Grabau J, Lane J, Steel-Goodwin L, et al. 1998. A subchronic exposure to trichloroethylene causes lipid peroxidation and hepatocellular proliferation in male B6C3F1 mouse liver. Toxicol Sci 43:145-154.

Chia S, Goh V, Ong C. 1997. Endocrine profiles of male workers with exposure to trichloroethylene. Am J Ind Med 32:217-222.

Chia SE, Ong CN, Tsakok MF, Ho A. 1996. Semen parameters in workers exposed to trichloroethylene. Reprod Toxicol 10:295-299.

Cosby N, Dukelow W. 1992. Toxicology of maternally ingested trichloroethylene (TCE) on embryonal and fetal development in mice and of TCE metabolites on in vitro fertilization. Toxicol Sci 19:268-274.

Crofton K, Lassiter T, Rebert C. 1994. Solvent-induced ototoxicity in rats: An atypical selective mid-frequency hearing deficit. Hear Res 80:25-30.

Crofton K, Zhao X. 1997. The ototoxicity of trichloroethylene: Extrapolation and relevance of high-concentration, short-duration animal exposure data. Fundam Appl Toxicol 38:101-106.

Cummings B, Lash L. 2000. Metabolism and toxicity of trichloroethylene and s-(1,2-dichlorovinyl)-l-cysteine in freshly isolated human proximal tubular cells. Toxicol Sci 53:458-466.

Cummings B, Lasker J, Lash L. 2000a. Expression of glutathione-dependent enzymes and cytochrome p450s in freshly isolated and primary cultures of proximal tubular cells from human kidney. J Pharmacol Exp Ther 293:677-685.

Cummings B, Zangar R, Novak R, Lash L. 2000b. Cytotoxicity of trichloroethylene and s-(1, 2-dichlorovinyl)-l-cysteine in primary cultures of rat renal proximal tubular and distal tubular cells. Toxicology 150:83-98.

Dai Y, Leng S, Li L, Niu Y, Huang H, Cheng J, et al. 2004. Genetic polymorphisms of cytokine genes and risk for trichloroethylene-induced severe generalized dermatitis: A case-control study. Biomarkers 9:470-478.

Davis S, Laszlo Pallos L, Wu J, Sapp J, Cusack C. 2005. Atsdr's trichloroethylene subregistry methods and results: 1989-2000. Arch Environ Occup Health 60:130-139.

Dawson B, Johnson P, Goldberg S, Ulreich J. 1990. Cardiac teratogenesis of trichloroethylene and dichloroethylene in a mammalian model. J Am Coll Cardiol 16:1304-1309.

Dawson B, Johnson P, Goldberg S, Ulreich J. 1993. Cardiac teratogenesis of halogenated hydrocarbon-contaminated drinking water. J Am Coll Cardiol 21:1466-1472.

Dees C, Travis C. 1993. The mitogenic potential of trichloroethylene in B6C3F1 mice. Toxicol Lett 69:129-137.

Diot E, Lesire V, Guilmot J, Metzger M, Pilore R, Rogier S, et al. 2002. Systemic sclerosis and occupational risk factors: A case-control study. Occup Environ Med 59:545-549.

Dorfmueller M, Henne S, York R, Bornschein R, Manson J. 1979. Evaluation of teratogenicity and behavioral toxicity with inhalation exposure of maternal rats to trichloroethylene. Toxicology 14:153-166.

Dow J, Green T. 2000. Trichloroethylene induced vitamin b(12) and folate deficiency leads to increased formic acid excretion in the rat. Toxicology 146:123-136.

Drake V, Koprowski S, Hu N, Smith S, Lough J. 2006a. Cardiogenic effects of trichloroethylene and trichloroacetic acid following exposure during heart specification of avian development. Toxicol Sci 94:153-162.

Drake V, Koprowski S, Lough J, Hu N, Smith S. 2006b. Trichloroethylene exposure during cardiac valvuloseptal morphogenesis alters cushion formation and cardiac hemodynamics in the avian embryo. Environ Health Perspect 114:842-847.

Driscoll T, Hamdan H, Wang G, Wright P, Stacey N. 1992. Concentrations of individual serum or plasma bile acids in workers exposed to chlorinated aliphatic hydrocarbons. Br J Ind Med 49:700-705.

DuTeaux S, Hengel M, DeGroot D, Jelks K, Miller M. 2003. Evidence for trichloroethylene bioactivation and adduct formation in the rat epididymis and efferent ducts. Biol Reprod 69:771-779.

DuTeaux S, Berger T, Hess R, Sartini B, Miller M. 2004. Male reproductive toxicity of trichloroethylene: Sperm protein oxidation and decreased fertilizing ability. Biol Reprod 70:1518-1526.

El Ghawabi S, Mansoor M, El Gamel M, El Saharti A, El Enany F. 1973. Chronic trichloroethylene exposure. J Egypt Med Assoc 56:715-724.

Elcombe C, Rose M, Pratt I. 1985. Biochemical, histological, and ultrastructural changes in rat and mouse liver following the administration of trichloroethylene: Possible relevance to species differences in hepatocarcinogenicity. Toxicol Appl Pharmacol 79:365-376.

Epstein D, Nolen G, Randall J, Christ S, Read E, Stober J, et al. 1992. Cardiopathic effects of dichloroacetate in the fetal long-evans rat. Teratology 46:225-235.

Fechter L, Liu Y, Herr D, Crofton K. 1998. Trichloroethylene ototoxicity: Evidence for a cochlear origin. Toxicol Sci 42:28-35.

Feldman R, Chirico-Post J, Proctor S. 1988. Blink reflex latency after exposure to trichloroethylene in well water. Arch Environ Health 43:143-148.

Feldman R, Niles C, Proctor S, Jabre J. 1992. Blink reflex measurement of effects of trichloroethylene exposure on the trigeminal nerve. Muscle Nerve 15:490-495.

Fisher J, Channel S, Eggers J, Johnson P, MacMahon K, Goodyear C, et al. 2001. Trichloroethylene, trichloroacetic acid, and dichloroacetic acid: Do they affect fetal rat heart development. Int J Toxicol 20:257-267.

Forkert P, Lash L, Nadeau V, Tardif R, Simmonds A. 2002. Metabolism and toxicity of trichloroethylene in epididymis and testis. Toxicol Appl Pharmacol 182:244-254.

Forkert P, Lash L, Tardif R, Tanphaichitr N, Vandevoort C, Moussa M. 2003. Identification of trichloroethylene and its metabolites in human seminal fluid of workers exposed to trichloroethylene. Drug Metab Dispos 31:306-311.

Fredriksson A, Danielsson B, Eriksson P. 1993. Altered behaviour in adult mice orally exposed to tri- and tetrachloroethylene as neonates. Toxicol Lett 66:13-19.

Gamberale G, Annwall G, Olson B. 1976. Exposure to trichloroethylene iii. Psychological functions. Scand J Work Environ Health 4:220-224.

Garabrant D, Lacey JV J, Laing T, Gillespie B, Mayes M, Cooper B, et al. 2003. Scleroderma and solvent exposure among women. Am J Epidemiol 157:493-500.

Gash D, Rutland K, Hudson N, Sullivan P, Bing G, Cass W, et al. 2008. Trichloroethylene: Parkinsonism and complex 1 mitochondrial neurotoxicity. Ann Neurol 63:184-192.

George J, Reel J, Myers C, Lawton A. 1985. Trichloroethylene: Reproduction and fertility assessment in cd-1 mice when administered in the feed. NTP-86-068. Research Triangle Park, NC:NIEHS.

George J, Reel J, Myers C, Lawton A, Lamb J, IV. 1986. Trichloroethylene: Reproduction and fertility assessment in F344 rats when administered in the feed. NTP-86-085. Research Triangle Park, NC:National Institute of Environmental Health Sciences, National Toxicology Program.

Gilkeson G, Keil D, Peden-Adams M. 2004. Immune effects of trichloroethylene on autoimmune disease in mice.87 - 98 PUBL- The Medical University of South Carolina Press.

Goel S, Rao G, Pandya K, Shanker R. 1992. Trichloroethylene toxicity in mice: A biochemical, hematological and pathological assessment. Indian J Exp Biol 30:402-406.

Goh V, Chia S, Ong C. 1998. Effects of chronic exposure to low doses of trichloroethylene on steroid hormone and insulin levels in normal men. Environ Health Perspect 106:41-44.

Goldberg S, Lebowitz M, Graver E, Hicks S. 1990. An association of human congenital cardiac malformations and drinking water contaminants. J Am Coll Cardiol 16:155-164.

Goldsworthy T, Popp J. 1987. Chlorinated hydrocarbon-induced peroxisomal enzyme activity in relation to species and organ carcinogenicity. Toxicol Appl Pharmacol 88:225-233.

Grandjean E, Munchinger R, Turrian V, Haas P, Knoepfel H-K, Rosenmund H. 1955. Investigations into the effects of exposure to trichlorethylene in mechanical engineering. Br J Ind Med 12:131-142.

Green T, Mainwaring G, Foster J. 1997. Trichloroethylene-induced mouse lung tumors: Studies of the mode of action and comparisons between species. Fundam Appl Toxicol 37:125-130.

Green T, Dow J, Foster J, Hext P. 1998. Formic acid excretion in rats exposed to trichloroethylene: A possible explanation for renal toxicity in long-term studies. Toxicology 127:39-47.

Green T, Dow J, Foster J. 2003. Increased formic acid excretion and the development of kidney toxicity in rats following chronic dosing with trichloroethanol, a major metabolite of trichloroethylene. Toxicology 191:109-119.

Green T, Dow J, Ong C, Ng V, Ong H, Zhuang Z, et al. 2004. Biological monitoring of kidney function among workers occupationally exposed to trichloroethylene. Occup Environ Med 61:312-317.

Griffin J, Blossom S, Jackson S, Gilbert K, Pumford N. 2000a. Trichloroethylene accelerates an autoimmune response by th1 t cell activation in mrl +/+ mice. Immunopharmacology 46:123-137.

Griffin J, Gilbert K, Lamps L, Pumford N. 2000b. Cd4+ t-cell activation and induction of autoimmune hepatitis following trichloroethylene treatment in mrl+/+ mice. Toxicol Sci 57:345-352.

Gun R, Grygorcewicz C, Nettelbeck T. 1978. Choice reaction time in workers using trichloroethylene. Med J Aust 1:535-536.

Hardin B, Bond G, Sikov M, Andrew F, Beliles R, Niemeier R. 1981. Testing of selected workplace chemicals for teratogenic potential. Scand J Work Environ Health 7:66-75.

Healy T, Poole T, Hopper A. 1982. Rat fetal development and maternal exposure to trichloroethylene 100 ppm. Br J Anaesth 54:337-341.

Hirsch A, Rankin K, Panelli P. 1996. Trichloroethylene exposure and headache. Headache Q 7:126-138.

Hobara T, Kobayashi H, Higashihara E, Kawamoto T, Sakai T. 1984. Acute effects of 1,1,1-trichloroethane, trichloroethylene, and toluene on the hematologic parameters in dogs. Arch Environ Contam Toxicol 13:589-593.

Huang H, Li L, Chen B, Huang J, Kuang S. 2002. New problems caused by occupational trichloroethylene exposure. Int J Immunopathol Pharmacol 15:30-32.

Iavicoli I, Marinaccio A, Carelli G. 2005. Effects of occupational trichloroethylene exposure on cytokine levels in workers. J Occup Environ Med 47:453-457.

Isaacson LG, Taylor DH. 1989. Maternal exposure to 1,1,2-trichloroethylene affects myelin in the hippocampal formation of the developing rat. Brain Research 488:403-407.

Isaacson LG, Spohler SA, Taylor DH. 1990. Trichloroethylene affects learning and decreases myelin in the rat hippocampus. Neurotoxicol Teratol 12:375-381.

Jacob S, Hery M, Protois J, Rossert J, Stengel B. 2007. New insight into solvent-related end-stage renal disease: Occupations, products and types of solvents at risk. Occup Environ Med 64:843–848.

Jaffe D, Gandolfi A, Nagle R. 1984. Chronic toxicity of s-(trans-1,2-dichlorovinyl)-l-cysteine in mice. J Appl Toxicol 4:315-319.

Jaspers R, Muijser H, Lammers J, Kulig B. 1993. Mid-frequency hearing loss and reduction of acoustic startle responding in rats following trichloroethylene exposure. Neurotoxicol Teratol 15:407-412.

Johnson P, Dawson B, Goldberg S. 1998. Cardiac teratogenicity of trichloroethylene metabolites. J Am Coll Cardiol 32:540-545.

Johnson P, Dawson B, Goldberg S. 1998. A review: Trichloroethylene metabolites: Potential cardiac teratogens. Environ Health Perspect 106 Suppl 4:995-999.

Johnson P, Goldberg S, Mays M, Dawson B. 2003. Threshold of trichloroethylene contamination in maternal drinking waters affecting fetal heart development in the rat. Environ Health Perspect 111:289-292.

Johnson P, Goldberg S, Mays M, Dawson B. 2005. Correction: Threshold of trichloroethylene contamination in maternal drinking waters affecting fetal heart development in the rat. Environ Health Perspect 113:A18.

Kamijima M, Hisanaga N, Wang H, Nakajima T. 2007. Occupational trichloroethylene exposure as a cause of idiosyncratic generalized skin disorders and accompanying hepatitis similar to drug hypersensitivities. Int Arch Occup Environ Health 80:357-370.

Kamijima M, Wang H, Huang H, Li L, Shibata E, Lin B, et al. 2008. Trichloroethylene causes generalized hypersensitivity skin disorders complicated by hepatitis. J Occup Health 50:328-338.

Kan FW, Forkert PG, Wade MG. 2007. Trichloroethylene exposure elicits damage in epididymal epithelium and spermatozoa in mice. Histol Histopathol 22:977-988.

Kaneko T, Saegusa M, Tasaka K, Sato A. 2000. Immunotoxicity of trichloroethylene: A study with mrl-lpr/lpr mice. J Appl Toxicol 20:471-475.

Kauffmann B, White K, Sanders V, Douglas K, Sain L, Borzelleca J, et al. 1982. Humoral and cell-mediated immune status in mice exposed to chloral hydrate. Environ Health Perspect 44:147-151.

Keil D, Peden-Adams M, Wallace S, Ruiz P, Gilkeson G. 2009. Assessment of trichloroethylene (TCE) exposure in murine strains genetically-prone and non-prone to develop autoimmune disease. J Environ Sci Health A Tox Hazard Subst Environ Eng 44:443-453.

Khan M, Kaphalia B, Prabhakar B, Kanz M, Ansari G. 1995. Trichloroethene-induced autoimmune response in female mrl +/+ mice. Toxicol Appl Pharmacol 134:155-160.

Kilburn K, Warshaw R. 1993. Effects on neurobehavioral performance of chronic exposure to chemically contaminated well water. Toxicol Ind Health 9:391-404.

Kilburn K, Thornton J. 1996. Prediction equations for simple and visual two-choice reactions times in environmental neurotoxicology. Arch Environ Health 51:439-444.

Kilburn K. 2002a. Is neurotoxicity associated with environmental trichloroethylene (TCE)? Arch Environ Health 57:113-120.

Kilburn K. 2002b. Do duration of exposure, proximity to electronic manufacturing plants, and involvement in a lawsuit affect chlorinated solvent toxicity. Arch Environ Health 57:121-126.

Kishi R, Harabuchi I, Ikeda T, Katakura Y, Miyake H. 1993. Acute effects of trichloroethylene on blood concentrations and performance decrements in rats and their relevance to humans. Occup Environ Med 50:470-480.

Kjellstrand P, Lanke J, Bjerkemo M, Zetterqvist L, Månsson L. 1980. Irreversible effects of trichloroethylene exposure on the central nervous system. Scand J Work Environ Health 6:40-47.

Kjellstrand P, Kanje M, Månsson L, Bjerkemo M, Mortensen I, Lanke J, et al. 1981. Trichloroethylene: Effects on body and organ weights in mice, rats and gerbils. Toxicology 21:105-115.

Kjellstrand P, Holmquist B, Alm P, Kanje M, Romare S, Jonsson I, et al. 1983a. Trichloroethylene: Further studies of the effects on body and organ weights and plasma butyrylcholinesterase activity in mice. Acta Pharmacol Toxicol 53:375-384.

Kjellstrand P, Holmquist B, Mandahl N, Bjerkemo M. 1983b. Effects of continuous trichloroethylene inhalation on different strains of mice. Acta Pharmacol Toxicol 53:369-374.

Kulig B. 1987. The effects of chronic trichloroethylene exposure on neurobehavioral functioning in the rat. Neurotoxicol Teratol 9:171-178.

Kumar P, Prasad A, Dutta K. 2000a. Steroidogenic alterations in testes and sera of rats exposed to trichloroethylene (TCE) by inhalation. Hum Exp Toxicol 19:117-121.

Kumar P, Prasad A, Saxena D, Manu U, Maji B, Dutta K. 2000b. Fertility and general reproduction studies in trichloroethylene exposed rats. Indian Journal of Occupational Health 43:117-126.

Kumar P, Prasad A, Maji B, Mani U, Dutta K. 2001a. Hepatoxic alterations induced by inhalation of trichlorethylene (TCE) in rats. Biomed Environ Sci 14:325-332.

Kumar P, Prasad A, Mani U, Maji B, Dutta K. 2001b. Trichloroethylene induced testicular toxicity in rats exposed by inhalation. Hum Exp Toxicol 20:585-589.

Kurasawa K. 1988. Selective damage of pulmonary nonciliated bronchiolar epithelial (Clara) cells by trichloroethylene in rats. Sangyo Eiseigaku Zasshi 30:121-133.

Lagakos S, Wessen B, Zelen M. 1986. An analysis of contaminated well water and health effects in woburn, massachusetts. J Am Stat Assoc 81:583-596.

Land P, Owen E, Linde H. 1981. Morphologic changes in mouse spermatozoa after exposure to inhalational anesthetics during early spermatogenesis. Anesthesiology 54:53-56.

Laughter A, Dunn C, Swanson C, Howroyd P, Cattley R, Corton J. 2004. Role of the peroxisome proliferator-activated receptor alpha (pparalpha) in responses to trichloroethylene and metabolites, trichloroacetate and dichloroacetate in mouse liver. Toxicology 203:83-98.

Le Mesurier S, Lykke A, Stewart B. 1980. Reduced yield of pulmonary surfactant: Patterns of response following administration of chemicals to rats by inhalation. Toxicol Lett 5:89-93.

Lehmann I, Rehwagen M, Diez U, Seiffart A, Rolle-Kampczyk U, Richter M, et al. 2001. Enhanced in vivo ige production and t cell polarization toward the type 2 phenotype in association with indoor exposure to voc: Results of the lars study. Int J Hyg Environ Health 204:211-221.

Lehmann I, Thoelke A, Rehwagen M, Rolle-Kampczyk U, Schlink U, Schulz R, et al. 2002. The influence of maternal exposure to volatile organic compounds on the cytokine secretion profile of neonatal t cells. Environ Toxicol 17:203-210.

Leigh J, Jiang W. 1993. Liver cirrhosis deaths within occupations and industries in the california occupational mortality study. Addiction 88:767-779.

Lewis G, Reynolds R, Johnson A. 1984. Some effects of trichloroethylene on mouse lungs and livers. Vascul Pharmacol 15:139-144.

Lindbohm M, Taskinen H, Sallmen M, Hemminki K. 1990. Spontaneous abortions among women exposed to organic solvents. Am J Ind Med 17:449-463.

Liu YT, Jin C, Chen Z, Cai SX, Yin SN, Li GL, et al. 1988. Increased subjective symptom prevalence among workers exposed to trichloroethylene at sub-oel levels. Tohoku J Exp Med 155:183-195.

Loeber C, Hendrix M, Diez De Pinos S, Goldberg S. 1988. Trichloroethylene: A cardiac teratogen in developing chick embryos. Pediatr Res 24:740-744.

Maitre A, Hours M, Bonneterre V, Arnaud J, Arslan M, Carpentier P, et al. 2004. Systemic sclerosis and occupational risk factors: Role of solvents and cleaning products. J Rheumatol 31:2395-2401.

Manson J, Murphy M, Richdale N, Smith M. 1984. Effects of oral exposure to trichloroethylene on female reproductive function. Toxicology 32:229-242.

Melnick R, Jameson C, Goehl T, Maronpot R, Collins B, Greenwell A, et al. 1987. Application of microencapsulation for toxicology studies: Ii. Toxicity of microencapsulated trichloroethylene in fischer 344 rats. Fundam Appl Toxicol 8:432-442.

Merrick B, Robinson M, Condie L. 1989. Differing hepatotoxicity and lethality after subacute trichloroethylene exposure in aqueous or corn oil gavage vehicles in B6C3F1 mice. J Appl Toxicol 9:15-21.

Mhiri C, Choyakh F, Ben Hmida M, Feki I, Ben Messaoud M, Zouari N. 2004. Trigeminal somatosensory evoked potentials in trichloroethylene-exposed workers. Neurosciences 9:102-107.

Mirsalis J, Tyson C, Steinmetz K, Loh E, Hamilton C, Bakke J, et al. 1989. Measurement of unscheduled DNA synthesis and s-phase synthesis in rodent hepatocytes following in vivo treatment: Testing of 24 compounds. Environ Mol Mutagen 14:155-164.

Mishima N, Hoffman S, Hill E, Krug E. 2006. Chick embryos exposed to trichloroethylene in an ex ovo culture model show selective defects in early endocardial cushion tissue formation. Birth Defects Res A Clin Mol Teratol 76:517-527.

Moser V, Cheek B, MacPhail R. 1995. A multidisciplinary approach to toxicological screening: Iii. Neurobehavioral toxicity. J Toxicol Environ Health A 45:173-210.

Moser V, MacPhail R, Gennings C. 2003. Neurobehavioral evaluations of mixtures of trichloroethylene, heptachlor, and di(2-ethylhexyl)phthlate in a full-factorial design. Toxicology 188:125-137.

Muijser H, Lammers J, Kullig B. 2000. Effects of exposure to trichloroethylene and noise on hearing in rats. Noise Health 2:57-66.

Nagaya T, Ishikawa N, Hata H. 1989. Urinary total protein and "beta"-2-microglobulin in workers exposed to trichloroethylene. Environ Res 50:86-92.

Nagaya T, Ishikawa N, Hata H, Otobe T. 1993. Subclinical and reversible hepatic effects of occupational exposure to trichloroethylene. Int Arch Occup Environ Health 64:561-563.

Nakajima T, Kamijo Y, Usuda N, Liang Y, Fukushima Y, Kametani K, et al. 2000. Sex-dependent regulation of hepatic peroxisome proliferation in mice by trichloroethylene via peroxisome proliferator-activated receptor alpha (pparalpha). Carcinogenesis 21:677-682.

Narotsky M, Kavlock R. 1995. A multidisciplinary approach to toxicological screening: Ii. Developmental toxicity. J Toxicol Environ Health 45:145-171.

Narotsky M, Weller E, Chinchilli V, Kavlock R. 1995. Nonadditive developmental toxicity in mixtures of trichloroethylene, di(2-ethylhexyl) phthalate, and heptachlor in a 5 x 5 x 5 design. Fundam Appl Toxicol 27:203-216.

NCI. 1976. Carcinogenesis bioassay of trichloroethylene. (Technical Report Series). NCI-CG-TR-2. Bethesda, MD:U.S. Department of Health, Education, and Welfare, Public Health Service, National Institutes of Health. Available: http://ntp.niehs.nih.gov/ntp/htdocs/LT_rpts/tr002.pdf.

Neghab M, Qu S, Bai C, Caples J, Stacey N. 1997. Raised concentration of serum bile acids following occupational exposure to halogenated solvents, 1,1,2-trichloro-1,2,2-trifluoroethane and trichloroethylene. Int Arch Occup Environ Health 70:187-194.

Nietert P, Sutherland S, Silver R, Pandey J, Knapp R, Hoel D, et al. 1998. Is occupational organic solvent exposure a risk factor for scleroderma? Arthritis Rheum 41:1111-1118.

Niklasson M, Tham R, Larsby B, Eriksson B. 1993. Effects of toluene, styrene, trichloroethylene, and trichloroethane on the vestibulo- and opto-oculo motor system in rats. Neurotoxicol Teratol 15:327-334.

NTP. 1988. Toxicology and carcinogenesis studies of trichloroethylene (cas no. 79-01-6) in four strains of rats (ACI, August, Marshall, Osborne-Mendel) (gavage studies). Research Triangle Park, NC:U.S. Department of Health and Human Services, Public Health Service, National Institutes of Health. Available: http://ntp.niehs.nih.gov/ntp/htdocs/LT_rpts/tr273.pdf.

NTP. 1990. Carcinogenesis studies of trichloroethylene (without epichlorohydrin) (cas no. 79-01-6) in F344/n rats and B6C3F1 mice (gavage studies). (Technical Report Series). Research Triangle Park, NC:U.S. Department of Health and Human Services, Public Health Service, National Institutes of Health. Available: http://ntp.niehs.nih.gov/ntp/htdocs/LT_rpts/tr243.pdf.

Nunes J, Ehrich M, Robertson J. 2001. Toxicosis associated with dual oral exposure of rats to lead and trichloroethylene. Toxicol Pathol 29:451-457.

Odum J, Foster J, Green T. 1992. A mechanism for the development of Clara cell lesions in the mouse lung after exposure to trichloroethylene. Chem Biol Interact 83:135-153.

Ohta M, Saito T, Saito K, Kurasaki M, Hosokawa T. 2001. Effect of trichloroethylene on spatiotemporal pattern of ltp in mouse hippocampal slices. Int J Neurosci 111:257-271.

Okino T, Nakajima T, Nakano M. 1991. Morphological and biochemical analyses of trichloroethylene hepatotoxicity: Differences in ethanol- and phenobarbital-pretreated rats. Toxicol Appl Pharmacol 108:379-389.

Oshiro W, Krantz Q, Bushnell P. 2004. A search for residual behavioral effects of trichloroethylene in rats exposed as young adults. Neurotoxicol Teratol 26:239-251.

Ou J, Ou Z, McCarver D, Hines R, Oldham K, Ackerman A, et al. 2003. Trichloroethylene decreases heat shock protein 90 interactions with endothelial nitric oxide synthase: Implications for endothelial cell proliferation. Toxicol Sci 73:90-97.

Peden-Adams M, Eudaly J, Heesemann L, Smythe J, Miller J, Gilkeson G, et al. 2006. Developmental immunotoxicity of trichloroethylene (TCE): Studies in B6C3F1 mice. J Environ Sci Health A Tox Hazard Subst Environ Eng 41:249-271.

Peden-Adams M, Eudaly J, Lee A, Miller J, Keil D, Gilkeson G. 2008. Lifetime exposure to trichloroethylene (TCE) does not accelerate autoimmune disease in mrl +/+ mice. J Environ Sci Health A Tox Hazard Subst Environ Eng 43:1402-1409.

Prendergast J, Jones R, Jenkins LJ, Siegel J. 1967. Effects on experimental animals of long-term inhalation of trichloroethylene, carbon tetrachloride, 1,1,1-trichloroethane, dichlorodifluoromethane, and 1,1-dichloroethylene. Toxicol Appl Pharmacol 10:270-289.

Radican L, Wartenberg D, Rhoads G, Schneider D, Wedeen R, Stewart P, et al. 2006. A retrospective occupational cohort study of end-stage renal disease in aircraft workers exposed to trichloroethylene and other hydrocarbons. J Occup Environ Med 48:1-12.

Ramdhan D, Kamijima M, Yamada N, Ito Y, Yanagiba Y, Nakamura D, et al. 2008. Molecular mechanism of trichloroethylene-induced hepatotoxicity mediated by cyp2e1. Toxicol Appl Pharmacol 231:300-307.

Ramdhan D, Kamijima M, Wang D, Ito Y, Naito H, Yanagiba Y, et al. 2010. Differential response to trichloroethylene-induced hepatosteatosis in wild-type and pparalpha-humanized mice. Environ Health Perspect 118:1557-1563.

Rasmussen K, Sabroe S. 1986. Neuropsychological symptoms among metal workers exposed to halogenated hydrocarbons. Scand J Public Health 14:161-168.

Rasmussen K, Sabroe S, Wohlert M, Ingerslev H, Kappel B, Nielsen J. 1988. A genotoxic study of metal workers exposed to trichloroethylene sperm parameters and chromosome aberrations in lymphocytes. Int Arch Occup Environ Health 60:419-423.

Rasmussen K, Arlien-Søborg P, Sabroe S. 1993a. Clinical neurological findings among metal degreasers exposed to chlorinated solvents. Acta Neurol Scand 87:200-204.

Rasmussen K, Brogren CH, Sabroe S. 1993. Subclinical affection of liver and kidney function and solvent exposure. Int Arch Occup Environ Health 64:445-448.

Rasmussen K, Jeppesen H, Sabroe S. 1993b. Solvent-induced chronic toxic encephalopathy. Am J Ind Med 23:779-792.

Rasmussen K, Jeppesen H, Sabroe S. 1993c. Psychometric tests for assessment of brain function after solvent exposure. Am J Ind Med 24:553-565.

Rebert C, Day V, Matteucci M, Pryor G. 1991. Sensory-evoked potentials in rats chronically exposed to trichloroethylene: Predominant auditory dysfunction. Neurotoxicol Teratol 13:83-90.

Rebert C, Boyes W, Pryor G, Svensgaard D, Kassay K, Gordon G, et al. 1993. Combined effects of solvents on the rat's auditory system: Styrene and trichloroethylene. Int J Psychophysiol 14:49-59.

Rebert C, Schwartz R, Svendsgaard D, Pryor G, Boyes W. 1995. Combined effects of paired solvents on the rat's auditory system. Toxicology 105:345-354.

Reif J, Burch J, Nuckols J, Metzger L, Ellington D, Anger W. 2003. Neurobehavioral effects of exposure to trichloroethylene through a municipal water supply. Environ Res 93:248-258.

Rodenbeck S, Sanderson L, Rene A. 2000. Maternal exposure to trichloroethylene in drinking water and birth-weight outcomes. Arch Environ Health 55:188-194.

Rufer E, Hacker T, Lough J, Smith S. 2008. Low-dose trichloroethylene exposure during valvuloseptal morphogenesis causes ventricular septal defects in hatched chicks. Toxicologist 102:314.

Rufer E, Hacker T, Flentke G, Drake V, Brody M, Lough J, et al. 2010. Altered cardiac function and ventricular septal defect in avian embryos exposed to low-dose trichloroethylene. Toxicol Sci 113:444-452.

Ruijten M, Verberk M, Sallé H. 1991. Nerve function in workers with long term exposure to trichloroethene. Br J Ind Med 48:87-92.

Sagawa K, Nishitani H, Kawai H, Kuge Y, Ikeda M. 1973. Transverse lesion of spinal cord after accidental exposure to trichloroethylene. Int Arch Arbeitsmed 31:257-264.

Saihan E, Burton J, Heaton K. 1978. A new syndrome with pigmentation, scleroderma, gynaecomastia, raynaud's phenomenon and peripheral neuropathy. Br J Dermatol 99:437-440.

Sallmen M, Lindbohm M, Anttila A, Kyyronen P, Taskinen H, Nykyri E, et al. 1998. Time to pregnancy among the wives of men exposed to organic solvents. Occup Environ Med 55:24-30.

Sallmén M, Lindbohm M, Kyyrönen P, Nykyri E, Anttila A, Taskinen H, et al. 1995. Reduced fertility among women exposed to organic solvents. Am J Ind Med 27:699-713.

Salvini M, Binaschi S, Riva M. 1971. Evaluation of the psychophysiological functions in humans exposed to trichloroethylene. Br J Ind Med 28:293-295.

Sanders V, Tucker A, White K, Jr, Kauffmann B, Hallett P, Carchman R, et al. 1982. Humoral and cell-mediated immune status in mice exposed to trichloroethylene in the drinking water. Toxicol Appl Pharmacol 62:358-368.

Savolainen H, Pfäffli P, Tengén M, Vainio H. 1977. Trichloroethylene and 1,1,1-trichloroethane: Effects on brain and liver after five days intermittent inhalation. Arch Toxicol 38:229-237.

Saygun M, Cakmak A, Ekici A, Pinar T, Bulcun E, Ulu N, et al. 2007. Five annual observations of respiratory findings in gun factory workers exposed to solvents. J Occup Environ Med 49:909-912.

Schwetz B, Leong B, Gehring P. 1975. The effect of maternally inhaled trichloroethylene, perchloroethylene, methyl chloroform, and methylene chloride on embryonal and fetal development in mice and rats. Toxicol Appl Pharmacol 32:84-96.

Scott J, Forkert P, Oulton M, Rasmusson M-G, Temple S, Fraser M, et al. 1988. Pulmonary toxicity of trichloroethylene: Induction of changes in surfactant phospholipids and phospholipase a2 activity in the mouse lung. Exp Mol Pathol 49:141-150.

Selden A, Hultberg B, Ulander A, Ahlborg G, Jr. 1993. Trichloroethylene exposure in vapour degreasing and the urinary excretion of n-acetyl-"beta"-d-glucosaminidase. Arch Toxicol 67:224-226.

Selgrade M, Gilmour M. 2010. Suppression of pulmonary host defenses and enhanced susceptibility to respiratory bacterial infection in mice following inhalation exposure to trichloroethylene and chloroform. J Immunotoxicol 7:350-356.

Shih CL, Chen HH, Chiu TH. 2001. Acute exposure to trichloroethylene differentially alters the susceptibility to chemoconvulsants in mice. Toxicology 162:35-42.

Smith G. 1970. The investigation of the mental effects of trichlorethylene. Ergonomics 13:580-586.

Smith M, Randall J, Read E, Stober J. 1989. Teratogenic activity of trichloroacetic acid in the rat. Teratology 40:445-451.

Smith M, Randall J, Read E, Stober J. 1992. Developmental toxicity of dichloroacetate in the rat. Teratology 46:217-223.

Stewart B, Le Mesurier S, Lykke A. 1979. Correlation of biochemical and morphological changes induced by chemical injury to the lung. Chem Biol Interact 26:321-338.

Stewart R, Dodd H, Gay H, Erley D. 1970. Experimental human exposure to trichloroethylene. Arch Environ Health 20:64-71.

Tang X, Li L, Huang J, Deng Y. 2002. Guinea pig maximization test for trichloroethylene and its metabolites. Biomed Environ Sci 15:113-118.

Tang X, Que B, Song X, Li S, Yang X, Wang H, et al. 2008. Characterization of liver injury associated with hypersensitive skin reactions induced by trichloroethylene in the guinea pig maximization test. J Occup Health 50:114-121.

Tao L, Yang S, Xie M, Kramer P, Pereira M. 2000. Effect of trichloroethylene and its metabolites, dichloroacetic acid and trichloroacetic acid, on the methylation and expression of c-jun and c-myc protooncogenes in mouse liver: Prevention by methionine. Toxicol Sci 54:399-407.

Taskinen H, Anttila A, Lindbohm ML, Sallmén M, Hemminki K. 1989. Spontaneous abortions and congenital malformations among the wives of men occupationally exposed to organic solvents. Scand J Work Environ Health 15:345-352.

Taskinen H, Kyyronen P, Hemminki K. 1994. Laboratory work and pregnancy outcome. J Occup Med 36:311-319.

Taylor D, Lagory K, Zaccaro D, Pfohl R, Laurie R. 1985. Effect of trichloroethylene on the exploratory and locomotor activity of rats exposed during development. Sci Total Environ 47:415-420.

Terracini B, Parker V. 1965. A pathological study on the toxicity of s-dichlorovinyl-l-cysteine. Food Cosmet Toxicol 3:67-74.

Tham R, Larsby B, Ödkvist L, Norlander B, Hydén D, Aschan G, et al. 1979. The influence of trichloroethylene and related drugs on the vestibular system. Acta Pharmacol Toxicol 44:336-342.

Tham R, Bunnfors I, Eriksson B, Larsby B, Lindgren S, Odkvist L. 1984. Vestibulo-ocular disturbances in rats exposed to organic solvents. Acta Pharmacol Toxicol 54:58-63.

Triebig G, Essing H, Schaller K, Valentin H. 1976. [biochemical and psychological examinations of trichloroenthylene exposed volunteers (author's transl)]. Zentralbl Bakteriol, Parasitenkd, Infektionskrankh Hyg, Abt 1: Orig, Reihe B 163:383-416.

Triebig G, Lehrl S, Kinzel W, Erzigkeit H, Galster J, Schaller K. 1977a. [psychopathometrical results of follow-up studies of trichloroethylene-exposed persons (author's transl)]. Zentralbl Bakteriol, Parasitenkd, Infektionskrankh Hyg, Abt 1: Orig, Reihe B 164:314-377.

Triebig G, Schaller K, Erzigkeit H, Valentin L. 1977b. [biochemical investigations and psychological studies of persons chronically exposed to trichloroethylene with regard to non-exposure intervals]. Int Arch Occup Environ Health 38:149-162.

Triebig G, Trautner P, Weltle D, Saure E, Valentin H. 1982. Untersuchungen zur neurotoxizität von arbeitsstoffen. Int Arch Occup Environ Health 51:25-34.

Triebig G, Bestler W, Baumeister P, Valentin H. 1983. [investigations on neurotoxicity of chemical substances at the workplace: Iv. Determination of the motor and sensory nerve conduction velocity in persons occupationally exposed to a mixture of organic solvents]. Int Arch Occup Environ Health 52:139-150.

Tröster A, Ruff R. 1990. Neuropsychological sequelae of exposure to the chlorinated hydrocarbon solvents trichloroethylene and trichloroethane. Arch Clin Neuropsychol 5:31-47.

Tucker A, Sanders V, Barnes D, Bradshaw T, White K, Jr, Sain L, et al. 1982. Toxicology of trichloroethylene in the mouse. Toxicol Appl Pharmacol 62:351-357.

Umezu T, Yonemoto J, Soma Y, Miura T. 1997. Behavioral effects of trichloroethylene and tetrachloroethylene in mice. Pharmacol Biochem Behav 58:665-671.

Veeramachaneni D, Palmer J, Amann R. 2001. Long-term effects on male reproduction of early exposure to common chemical contaminants in drinking water. Hum Reprod 16:979-987.

Vernon R, Ferguson R. 1969. Effects of trichloroethylene on visual-motor performance. Arch Environ Occup Health 18:894-900.

Villaschi S, Giovanetti A, Lombardi C, Nicolai G, Garbati M, Andreozzi U. 1991. Damage and repair of mouse bronchial epithelium following acute inhalation of trichloroethylene. Exp Lung Res 17:601-614.

Wang G, Stacey N. 1990. Elevation of individual serum bile acids on exposure to trichloroethylene or alpha-naphthylisothiocyanate. Toxicol Appl Pharmacol 105:209-215.

Wang G, Cai P, Ansari G, Khan M. 2007. Oxidative and nitrosative stress in trichloroethene-mediated autoimmune response. Toxicology 229:186-193.

Wang G, König R, Ansari G, Khan M. 2008. Lipid peroxidation-derived aldehyde-protein adducts contribute to trichloroethene-mediated autoimmunity via activation of cd4+ t cells. Free Radic Biol Med 44:1475-1482.

Warren D, Graeter L, Channel S, Eggers J, Goodyear C, Macmahon K, et al. 2006. Trichloroethylene, trichloroacetic acid, and dichloroacetic acid: Do they affect eye development in the sprague-dawley rat. Int J Toxicol 25:279-284.

Waseem M, Ali M, Dogra S, Dutta K, Kaw J. 2001. Toxicity of trichloroethylene following inhalation and drinking contaminated water. J Appl Toxicol 21:441-444.

White K, David D, Butterworth L, Klykken P. 2000. Assessment of autoimmunity-inducing potential using the brown norway rat challenge model. Toxicol Lett 112-113:443-451.

White R, Feldman R, Eviator I, Jabre J, Niles C. 1997. Hazardous waste and neurobehavioral effects: A developmental perspective. Environ Res 73:113-124.

Windham G, Shusterman D, Swan S, Fenster L, Eskenazi B. 1991. Exposure to organic solvents and adverse pregnancy outcome. Am J Ind Med 20:241-259.

Wolff D, Siegmund R. 1978. [the circadian dependent effect of trichloroethylene on spontaneous locomotor activity and of tetrachloroethane on mortality in mice]. Biologisches Zentralblatt 97:345-351.

Woolhiser M, Krieger S, Thomas J, Hotchkiss J. 2006. Trichloroethylene (TCE): Immunotoxicity potential in cd rats following a 4-week vapor inhalation exposure. Midland, MI:Unpublished.

Wu K, Berger T. 2007. Trichloroethylene metabolism in the rat ovary reduces oocyte fertilizability. Chem Biol Interact 170:20-30.

Xu H, Tanphaichitr N, Forkert PG, Anupriwan A, Weerachatyanukul W, Vincent R, et al. 2004. Exposure to trichloroethylene and its metabolites causes impairment of sperm fertilizing ability in mice. Toxicol Sci 82:590-597.

Xu X, Yang R, Wu N, Zhong P, Ke Y, Zhou L, et al. 2009. Severe hypersensitivity dermatitis and liver dysfunction induced by occupational exposure to trichloroethylene. Ind Health 47:107-112.

Yamamura K, Ikeda T, Sadmoto T, Maehara N, Harabuchi I, Takashima H, et al. 1983. Effects of trichloroethylene exposure on hearing: An investigation of cochlear microphonics and action potential of the guinea pig. Eur J Appl Physiol 52:47-50.

Yauck J, Malloy M, Blair K, Simpson P, McCarver D. 2004. Proximity of residence to trichloroethylene-emitting sites and increased risk of offspring congenital heart defects among older women. Birth Defects Res A Clin Mol Teratol 70:808-814.

Zenick H, Blackburn K, Hope E, Richdale N, Smith M. 1984. Effects of trichloroethylene exposure on male reproductive function in rats. Toxicology 31:237-250.

Zielinski A. 1973. General health state of women professionally exposed to trichloroethylene vapours. Med Pr 24:263-271.
